# Inhibition of Fast Axonal Transport by Pathogenic SOD1 Involves Activation of p38 MAP Kinase

**DOI:** 10.1371/journal.pone.0065235

**Published:** 2013-06-12

**Authors:** Gerardo A. Morfini, Daryl A. Bosco, Hannah Brown, Rodolfo Gatto, Agnieszka Kaminska, Yuyu Song, Linda Molla, Lisa Baker, M. Natalia Marangoni, Sarah Berth, Ehsan Tavassoli, Carolina Bagnato, Ashutosh Tiwari, Lawrence J. Hayward, Gustavo F. Pigino, D. Martin Watterson, Chun-Fang Huang, Gary Banker, Robert H. Brown, Scott T. Brady

**Affiliations:** 1 Depart of Anatomy and Cell Biology, University of Illinois at Chicago, Chicago, Illinois, United States of America; 2 Marine Biological Laboratory, Woods Hole, Massachusetts, United States of America; 3 Department of Neurology, University of Massachusetts Medical Center, Worcester, Massachusetts, United States of America; 4 Department of Chemistry, Michigan Technological University, Houghton, Michigan, United States of America; 5 Department of Psychiatry, Massachusetts General Hospital, Boston, Massachusetts, United States of America; 6 Center for Molecular Innovation and Drug Discovery and Department of Molecular Pharmacology & Biological Chemistry, Northwestern University, Chicago, IIllinois, United States of America; 7 The Jungers Center for Neurosciences Research, Oregon Health & Science University, Portland, Oregon, United States of America; 8 Department of Natural Sciences and Engineering. National University of Rio Negro, Rio Negro, Argentina; University of Florida, United States of America

## Abstract

Dying-back degeneration of motor neuron axons represents an established feature of familial amyotrophic lateral sclerosis (FALS) associated with superoxide dismutase 1 (SOD1) mutations, but axon-autonomous effects of pathogenic SOD1 remained undefined. Characteristics of motor neurons affected in FALS include abnormal kinase activation, aberrant neurofilament phosphorylation, and fast axonal transport (FAT) deficits, but functional relationships among these pathogenic events were unclear. Experiments in isolated squid axoplasm reveal that FALS-related SOD1 mutant polypeptides inhibit FAT through a mechanism involving a p38 mitogen activated protein kinase pathway. Mutant SOD1 activated neuronal p38 in mouse spinal cord, neuroblastoma cells and squid axoplasm. Active p38 MAP kinase phosphorylated kinesin-1, and this phosphorylation event inhibited kinesin-1. Finally, vesicle motility assays revealed previously unrecognized, isoform-specific effects of p38 on FAT. Axon-autonomous activation of the p38 pathway represents a novel gain of toxic function for FALS-linked SOD1 proteins consistent with the dying-back pattern of neurodegeneration characteristic of ALS.

## Introduction

Amyotrophic lateral sclerosis (ALS) is a progressive, adult-onset, neurodegenerative disease mainly affecting function and survival of motor neurons [1]. Most ALS cases are sporadic (SALS) with no identified genetic defect, but 5–10% result from mutations in specific alleles causing familial forms of ALS (FALS). Genes associated with FALS encode proteins of diverse structure and function, including superoxide dismutase 1 (SOD1; reduction of superoxide radicals and redox signaling), an intronic expansion in the gene C9orf72 [2], [3], nucleic acid binding proteins TDP-43 and FUS/TLS [4], VAPB (vesicle trafficking), senataxin (helicase), and dynactin (cytoplasmic dynein accessory protein). Clinical phenotypes of SALS closely resemble FALS variants, suggesting possible overlapping pathogenic pathways between FALS and SALS [1].

Mutations in the SOD1 gene are the best-characterized cause of FALS [5]. Genetic evidence indicates that pathogenic effects of mutant SOD1 (mSOD1) reflect a toxic gain of function, but aspects relevant to ALS were difficult to identify, because mSOD1 can have multiple adverse effects on motor neurons [1]. Although motor neuron death represents the final outcome of ALS, pathological observations from ALS patients and transgenic mice expressing SOD1 mutants indicate that altered synaptic and axonal function occur much earlier than cell death, consistent with the “dying-back” pattern common to distal axonopathies [6]. However, little is known about molecular mechanisms underlying axonal degeneration in ALS [7]. Most FALS models rely on overexpression of mSOD1, making it impossible to distinguish pathogenic events in axonal compartment from those in neuronal cell bodies [7]. A major challenge in ALS research is the identification of disease-specific effects of mSOD1 in axons.

Transgenic expression of mSOD1 in mice (G93A-SOD1 mice) triggers motor neuron disease closely resembling human ALS [8], despite normal levels of endogenous SOD1. Pathological changes in motor neurons of mSOD1 transgenic mice include abnormal activation of protein kinases [9], [10], increased neurofilament phosphorylation [11], synaptic function abnormalities [12] and deficits in fast axonal transport (FAT) [13]–[15]. However, relevant pathogenic targets for activated kinases were not identified, and mechanisms linking kinase activation to axonal and synaptic degeneration were unknown.

We report here that pathogenic mSOD1 selectively inhibited fast axonal transport (FAT) in isolated axoplasm by a mechanism involving activation of axonal p38 mitogen-activated protein kinases (MAPKs) and kinesin-1 phosphorylation. Accordingly, activation of p38 MAPK was also observed in spinal cord of G93A-SOD1 mice. Moreover, detection of p38-phosphorylated serines 175/176 within kinesin-1, which impaired translocation of kinesin-1 along axonal microtubules, provides a molecular basis for inhibition of FAT by mSOD1. Axon-autonomous effects of mSOD1 provide a mechanism linking increased kinase activity, heightened neurofilament phosphorylation, and FAT deficits in FALS. Activation of the p38 MAPK pathway and consequent inhibition of FAT represents a toxic gain of function mechanism associated with pathogenic forms of SOD1.

## Results

### Pathogenic SOD1 mutant proteins selectively inhibit anterograde fast axonal transport

Although the axonal compartment is a critical pathogenic target in ALS [6], axon-specific effects of mSOD1 have not been previously defined [7]. Deficits in both anterograde and retrograde FAT were reported in FALS [16], [17], but the diverse effects of mSOD1 on gene transcription, microglial activation and apoptotic pathways left unclear whether these FAT deficits were a consequence or cause of these events [5], [18]. To evaluate axon-autonomous effects of pathogenic SOD1 we assayed FAT in isolated squid axoplasm, which lacks transcription, protein synthesis machinery, and glial components [19]. This *ex vivo* experimental system allows quantitative analysis of FAT for membrane-bounded organelles (MBOs) in both anterograde (conventional kinesin-dependent) and retrograde (cytoplasmic dynein-dependent) directions. The lack of plasma membrane in the isolated axoplasm preparation facilitates quantitative evaluation of how pathogenic proteins affect FAT [20].

**Figure 1:**
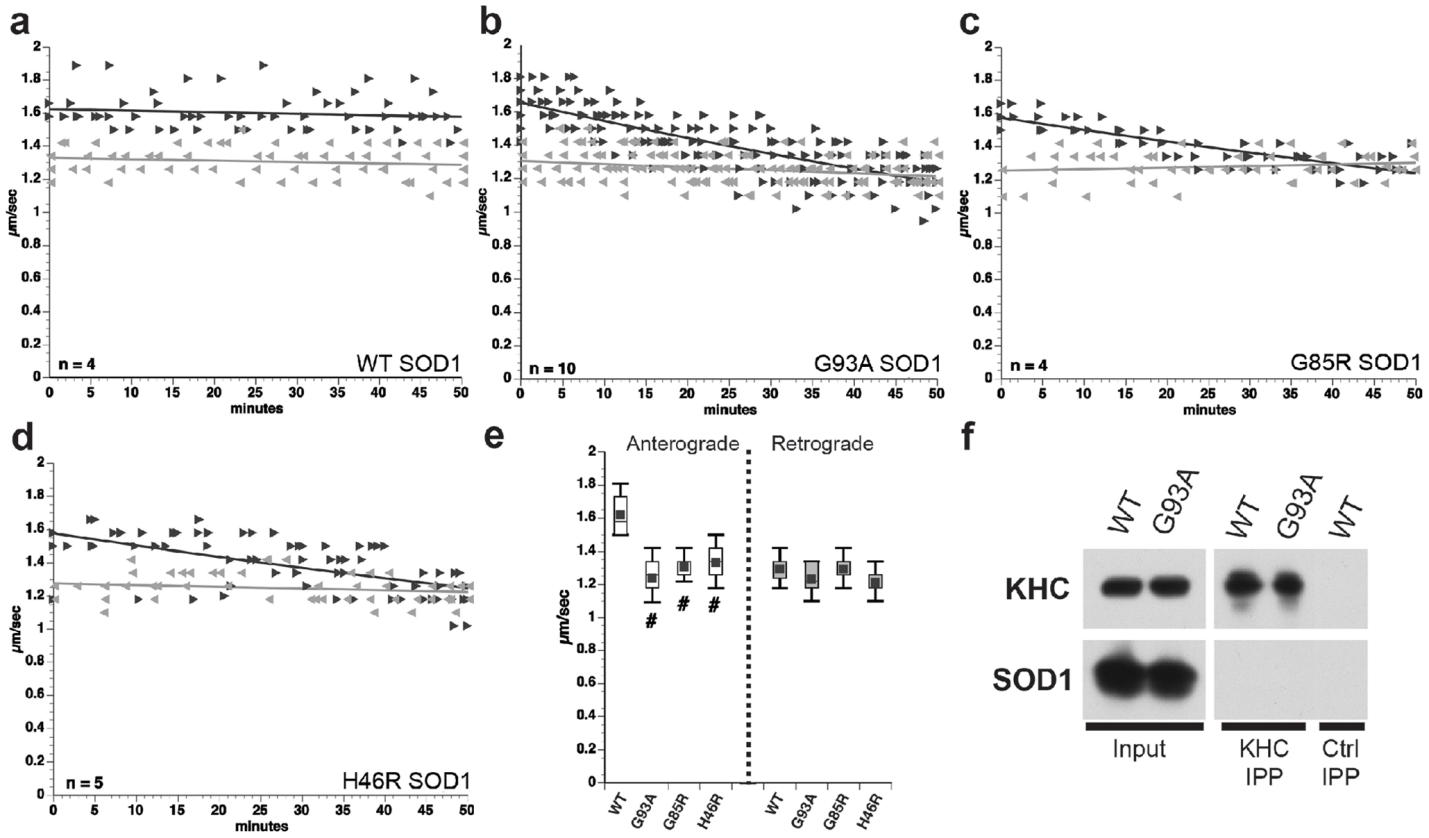
FALS-linked mutant SOD1 proteins selectively inhibit anterograde, conventional kinesin-dependent FAT. **(a–d)** Vesicle motility assays in isolated squid axoplasm. Individual velocity (µm/sec) rate measurements (arrowheads) are plotted as a function of time (minutes). Dark arrowheads and lines represent anterograde, conventional kinesin-dependent rates. Grey arrows and lines represent retrograde, cytoplasmic dynein-dependent rates. Perfusion of WT-SOD1 protein **(a)** in axoplasm shows no effect on either direction. In contrast, perfusion of G93A-SOD1 specifically inhibits anterograde, but not retrograde FAT **(b)**. Similar results were obtained after perfusion of SOD1-G85R **(c)** and SOD1-H46R **(d)**. *n*: number of experiments. (**e**) 30 to 50 minutes after perfusion, anterograde FAT rates were significantly lower in axoplasms perfused with mSOD1, than in axoplasms perfused with either WT-SOD1 (#: p≤0.01) or control buffer (not shown). All pathogenic SOD1 proteins tested had no effect on retrograde FAT. (**f**) Kinesin-1 (KHC IPP) was immunoprecipitated from spinal cord lysates of transgenic mice expressing either WT-SOD1 (WT) or G93A-SOD1 (G93A). An aliquot of lysate (Input) was included as positive control. Immunoblots using anti-kinesin-1 antibody (KHC) demonstrated effective immunoprecipitation from lysates, regardless of genotype. Specificity was confirmed by the absence of kinesin-1 on control immunoprecipitates with non-immune mouse IgG (Ctrl IPP). Immunoblot with an anti-SOD1 monoclonal antibody (D3H5) failed to detect SOD1 in kinesin-1 immunoprecipitates, suggesting that neither WT SOD1 nor G93A-SOD1 interact directly with kinesin-1.

Purified (>95%), recombinant wild type SOD1 (WT-SOD1) and three FALS-linked SOD1 mutants (G93A, G85R, and H46R) were prepared as described previously [21]. SOD1 was perfused at 5 µM and FAT analyzed by video-enhanced microscopy for 50 minutes (Fig. 1). SOD1 is an abundant protein in mammalian brain (present at µM levels) [22]. Although 5 µM was used standardly to facilitate comparisons, similar effects were observed with lower concentrations of pathogenic SOD1 (0.1–0.4 µM, not shown) [23]. Perfusion of WT-SOD1 had no effect on either direction of FAT (Fig. 1a). In contrast, perfusion of G93A-SOD1 selectively inhibited anterograde FAT (Fig. 1b). Similar results were obtained with G85R-SOD1 and H46R-SOD1 mutant variants (Figs. 1c–d). Quantitative analysis indicated that all mSOD1 proteins tested significantly inhibited anterograde, but not retrograde FAT (Fig. 1e). Selective inhibition of anterograde FAT by mSOD1 proteins ruled out effects on microtubule integrity, steric interference by potential SOD1 aggregates and ATP deficits, which would compromise *both* anterograde and retrograde transport.

To evaluate possible physical interactions of conventional kinesin with mSOD1, co-immunoprecipitation studies were undertaken [24]–[26]. Spinal cord lysates from transgenic mice expressing wild type SOD1 (WT-SOD1 mice) or G93A-SOD1 mice were compared [27]. Conventional kinesin is a heterotetramer composed of two heavy chains (kinesin-1, KHC) and two light chains (KLC) [24]. Antibodies recognizing KHCs effectively immunoprecipitated the conventional kinesin holoenzyme from both WT-SOD1 and G93A-SOD1 mouse spinal cord lysates (Fig. 1f). Under these conditions (see methods), KLCs co-precipitated with KHC (kinesin-1) [24]–[26]. However. anti-SOD1 antibodies failed to detect SOD1 in kinesin-1 immunoprecipitates. The lack of co-immunoprecipitation indicated that inhibitory effects of mSOD1 proteins on anterograde FAT did not result from direct interactions between mSOD1 and conventional kinesin, consistent with previous reports [28].

### Pathogenic SOD1 increases phosphorylation of neurofilaments and kinesin subunits in axoplasm

**Figure 2:**
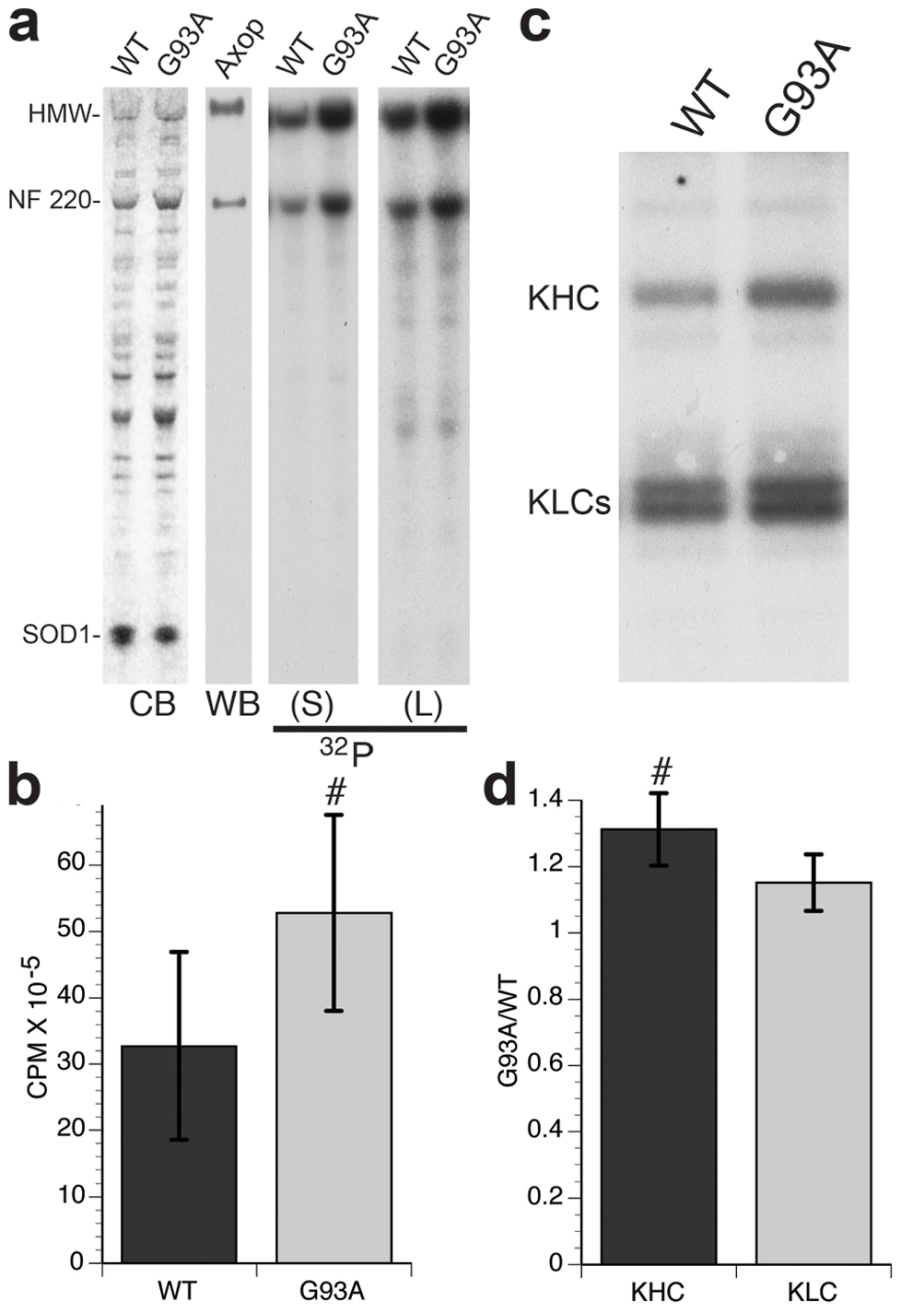
Pathogenic SOD1 increases neurofilament phosphorylation. Phosphorylation of squid neurofilaments (NF) in isolated “sister” axoplasms (see Methods) was analyzed using metabolic labeling experiments with ^32^P-γ-ATP. (**a)** Coomassie Blue staining (CB) shows similar levels of perfused WT-SOD1, G93A-SOD1 and total axoplasmic proteins. Immunoblot analysis (WB) with the NFH antibody SMI-31 confirmed the identity of major phosphorylated bands as NF220 and HMW, major NF subunits in squid axoplasm [30]. Short (S) and long (L) exposure of autoradiograms (^32^P) show increased phosphorylation of NF220 and HMW NF subunits in axoplasms perfused with G93A-SOD1, compared to WT-SOD1. **(b)** Quantitation of squid NF phosphorylation showed ⋍70% increase in G93A-SOD1 treated axoplasms, compared to those treated with WT-SOD1 (p≤0.01 (#) in a paired t-test). **(c)** In parallel experiments, kinesin-1 was immunoprecipitated from axoplasms labeled with γ-^32^P-ATP in the presence of WT-SOD1 or G93A-SOD1. Both heavy (KHC) and light (KLC) chains of conventional kinesin were phosphorylated. **(d)** The ratio of counts for G93A-SOD1/WT-SOD1 indicates that KHC labeling increased 31% in G93A-SOD1 axoplasms, compared to WT-SOD1 (significant at p≤0.05 by paired t-test, #). KLC phosphorylation increased by 15%, but was not statistically significant (p = 0.123). n = 7.

Our previous work showed that mutant proteins associated with neurodegenerative diseases can affect FAT by activating axonal kinases involved in the regulation of FAT [29]. Moreover, various studies reported abnormal activation of protein kinases in spinal cords of ALS patients and ALS mouse models [9], but relevant phosphorylated targets and pathways mediating kinase activation were not defined. To determine whether mSOD1 activated kinases, we performed metabolic labeling in isolated axoplasm. To control for variation in basal kinase activity, two “sister” axons were dissected from each squid and extruded (Fig. S1). Labeling with γ–^32^P-ATP, one axon was perfused with WT-SOD1 and the contralateral “sister” axon with G93A-SOD1. After 50 min, axoplasms were analyzed by SDS-PAGE and autoradiography (Fig. 2a), revealing two predominant ^32^P-labelled polypeptides at ≈220 kDa and >400 kDa. An antibody against mammalian NFH (SMI31) that recognizes squid neurofilaments confirmed identification of these bands as NF220 and high molecular weight NF aggregates (HMW) [30]. NF220 is a major phosphoprotein in squid axoplasm, homologous to mammalian NF heavy chain (NFH) [31]. Consistent with reports of increased NFH phosphorylation early in ALS [11], [32], HMW and NF220 phosphorylation increased in axoplasms perfused with G93A-SOD1, compared to WT-SOD1 (Fig. 2a). Phosphorimager analysis revealed an approximately 2-fold increase in overall NF220 phosphorylation (n = 4; p≤0.0284 in a paired t-test; Fig. 2b). The effect required structural integrity of axoplasms, because adding mSOD1 and ^32^P-ATP to homogenized axoplasms did not affect NF220 phosphorylation (not shown). This observation was consistent with previous observations that mechanical disruption of the axoplasm and nerve tissue alters the activity of kinases, phosphatases and chaperones, presumably due to altered compartmentation/organization of the various components [33].

To determine effects of mSOD1 on kinesin-1 phosphorylation, “sister” axoplasms were perfused with WT-SOD1 or G93A-SOD1and γ−^32^P-ATP (Fig. S1). Kinesin-1 was immunoprecipitated from axoplasm lysates, and incorporation of radiolabeled P^32^ analyzed by Phosphorimager [34]. Both KHC and KLC subunits of conventional kinesin incorporated ^32^P (Fig. 2c) [35]–[38]. Significantly, kinesin-1 phosphorylation increased by approximately 30% in axoplasms perfused with G93A-SOD1, relative to WT-SOD1 (Fig. 2d) without statistically significant changes in KLC phosphorylation. This increase likely represents an underestimation of changes at a given phosphorylation site, since some kinesin-1 residues are constitutively phosphorylated [38], [39]. Together, these experiments indicated that mSOD1 proteins increased NF and kinesin-1 (KHC) phosphorylation through activation of endogenous axonal kinases.

### p38 MAPK mediates inhibition of FAT induced by pathogenic SOD1

To identify specific kinases involved, we co-perfused axoplasms with G93A-SOD1 and specific inhibitors of NF kinases, including GSK3 and MAP kinases [40]. Previously, these approaches allowed identification of kinase-dependent pathogenic pathways activated by other neuropathogenic proteins. For example, selective inhibition of anterograde FAT by pathogenic tau is prevented by co-perfusion with GSK3 inhibitors [41], but effects of pathogenic androgen receptor [34] and huntingtin [26] on FAT are blocked by JNK inhibitors.

**Figure 3:**
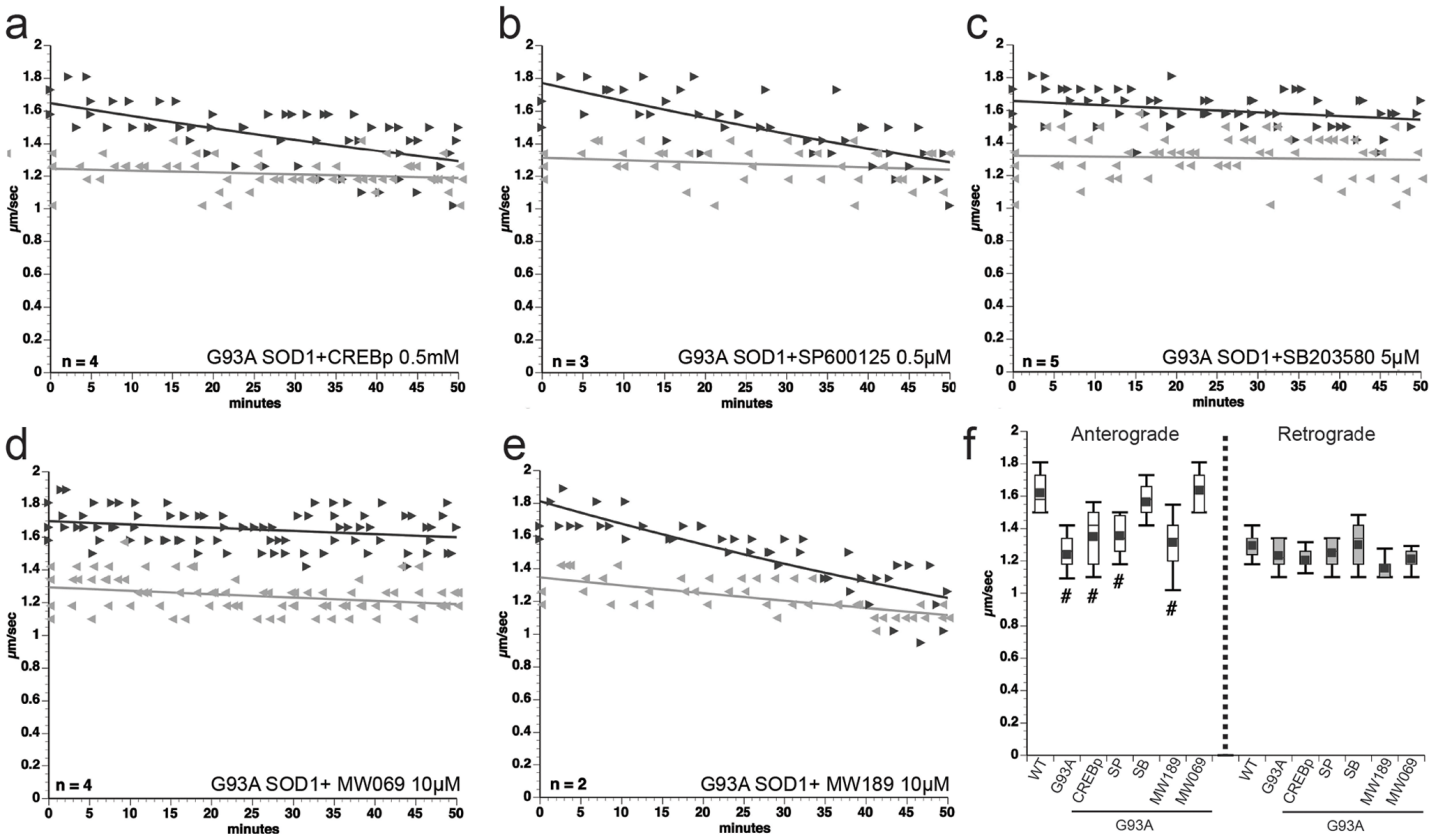
p38 MAPK mediates the inhibition of anterograde FAT by pathogenic SOD1. **(a–e)** Co-perfusion of G93A-SOD1 with either CREBp (a competitive inhibitor of GSK3) (**a**) or SP600125 (a pharmacological inhibitor of JNK) (**b**) did not block effects of G93A-SOD1 on anterograde FAT, but co-perfusion with SB203580 (an inhibitor of p38 and JNK2/3) completely blocked effects of mutant G93A-SOD1 on anterograde FAT **(c)**. MW01-2-069SRM (MW069) [45], a selective inhibitor of p38α structurally unrelated to SB203580 also blocked inhibition of FAT **(d),** but an inactive MW069 analog MW01-6-189WH (MW189) did not **(e)**. n = number of experiments. **(f)** Quantitation of **a–e** shows that SB203580 (SB) and MW069 compounds, but not CREBp, SP600125 (SP) or MW189 compounds blocked the effects of G93A-SOD1 on anterograde FAT (# indicates different from WT SOD1 at p≤0.001 by pooled t-test). Plots show the mean, standard deviation, maximum and minimum of FAT rates recorded 30–50 min for each experimental condition.

Co-perfusion of G93A-SOD1 with either CREBp (a competitive inhibitor of GSK3) [38] or SP600125 (an inhibitor of JNK kinases) [42] (Fig. 3a, b) failed to block mSOD1 effects on anterograde FAT. In contrast, SB203580 completely protected (Fig. 3c). SB203580 is a selective inhibitor of p38 MAPK [43] and JNK2/3 [44]. Effects of mSOD1 on anterograde FAT was also prevented by MW01-2-069SRM (MW069), a highly selective pharmacological inhibitor of p38 structurally unrelated to SB203580 [45] (Fig. 3d). In contrast, MW01-6-189WH (MW189), a structural analog of MW069 that is inactive as a p38 MAPK inhibitor, did not protect (Fig. 3e). Quantitative analysis showed that anterograde FAT rates for axoplasms co-perfused with G93A-SOD1 and either CREBp, SP600125, or MW189 were significantly lower than in axoplasms with WT-SOD1. Anterograde FAT rates for axoplasms with G93A-SOD1 and either SB203580 or MW069 were indistinguishable from controls (Fig. 3f). These data indicate that inhibition of anterograde FAT by mSOD1 mutants depends upon activation of axonal p38 in the axoplasm preparation.

**Figure 4:**
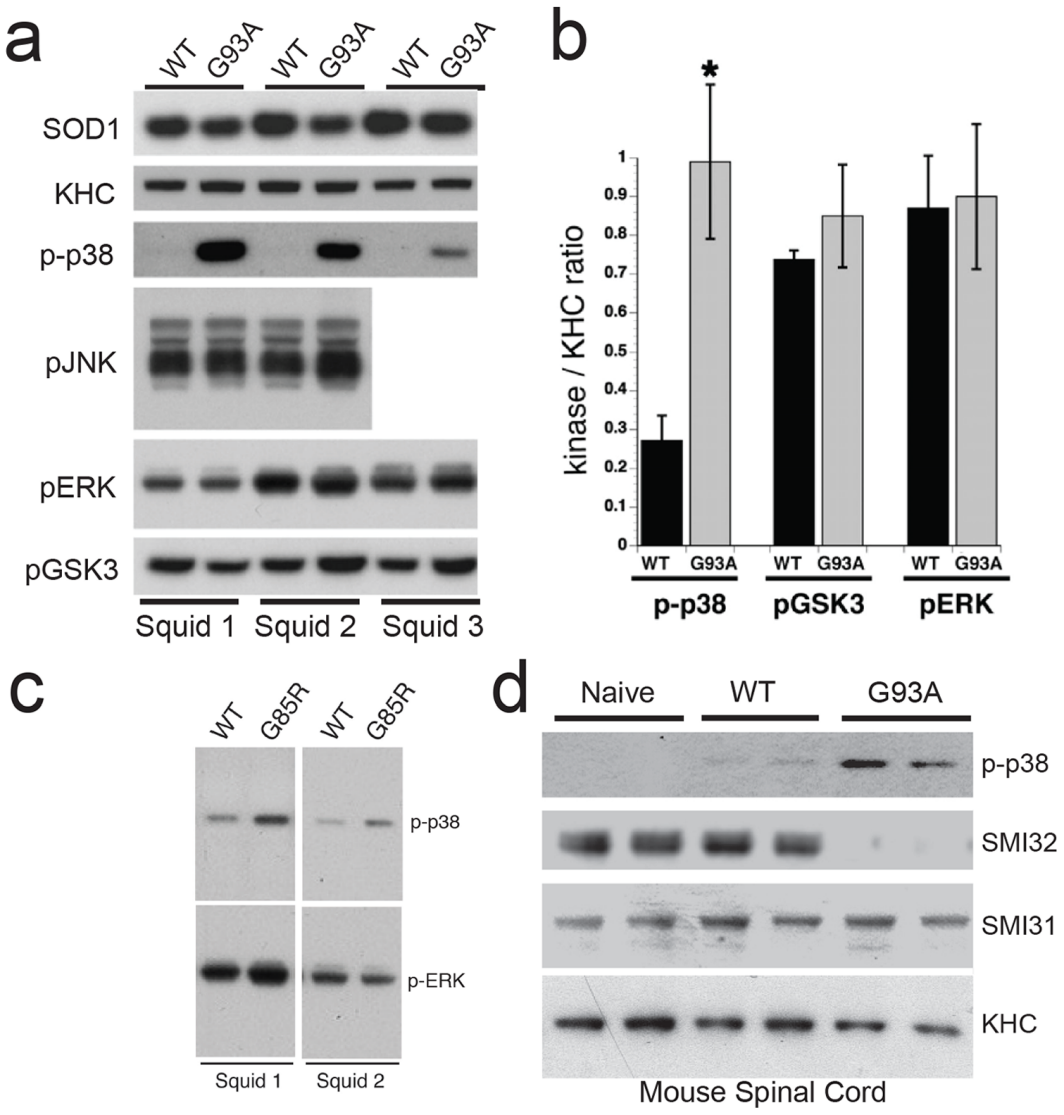
Activation of p38 MAPK by pathogenic SOD1 in axoplasm and mouse spinal cord. **(a)** Immunoblots with phosphoantibodies show activation of p38 (p-p38) in axoplasms perfused with G93A-SOD1 (G93A), compared to WT-SOD1 (WT). No changes were found in activities of GSK3 (p-GSK3), JNK (p-JNK) or ERK (p-ERK) with perfusion of SOD1. An SOD1 antibody confirmed that similar levels of WT-SOD1 and G93A-SOD1 were perfused, and kinesin-1 (KHC) antibodies serve as a loading control for axoplasmic protein. Representative results from three independent experiments (Squids 1–3) are shown. **(b)** Quantitation of blots reveals a 3–4 fold increase in p-p38 with G93A SOD1, compared to WT-SOD1 (*n* = 8; p≤0.05 (#) by a t-test). No significant differences were found in levels of activated GSK3 (*n* = 3) or ERK (*n* = 3) **(c)** Increased activation of p38, but not ERK was also seen in axoplasms perfused with G85R-SOD1 (G85R) polypeptides. **(d)** Phosphorylation of both neurofilaments (NF) and p38 MAPK was analyzed in spinal cords of age-matched (50 days old) non-transgenic (Naïve), human WT-SOD1 (WT) transgenic and human G93A-SOD1 (G93A) transgenic mice using phosphorylation sensitive antibodies. Kinesin heavy chain (KHC) blots show similar levels of protein loading. SMI32 antibodies recognize a dephosphorylated epitope in NFH that can be phosphorylated by MAPKs, whereas SMI31 recognizes an epitope not affected by phosphorylation with MAPKs [34]. SMI31 immunoreactivity showed similar levels in all mice and serves as a second loading control. In contrast, SMI32 reactivity is reduced in G93A-SOD1 mice, but not naïve and WT-SOD1 mice, suggesting increased phosphorylation of NFs by MAPKs in FALS. Accordingly, p38 activity (p-p38) was increased in spinal cord of G93A-SOD1 mice, with a slight activation in WT-SOD1 mice.

We also evaluated activation of various NF kinases in isolated axons using biochemical methods. “Sister” axoplasms were perfused with WT-SOD1 or G93A-SOD1 (Fig. S1). After 50 min, axoplasms were analyzed by immunoblotting. Antibodies recognizing phosphorylated forms of GSK3 (Ser9), ERK (Thr202/Tyr204) and JNK (Thr183/Tyr185) showed no differences in activity between WT-SOD1 and G93A-SOD1-perfused axoplasms (Fig. 4a). In contrast, antibodies against active p38 phosphorylated at Thr180/Tyr182 (p-p38), revealed increased p38 activity in axoplasms perfused with G93A-SOD1, compared to WT-SOD1 (Fig. 4a). G93A-SOD1 induced an approximately 4-fold increase in p38 activation, as compared to WT-SOD1 (*n = *8; Fig. 4b). Increased p38 phosphorylation was also seen with G85R-SOD1-perfused axoplasms (Fig. 4c) [46]. Effects of mSOD1 proteins on p38 activity were consistent with those induced by an oxidized form of WT-SOD1, which mimics toxic properties of FALS-linked SOD1 mutants [23].

To validate these observations in mammalian FALS-affected neurons, we examined both NF phosphorylation and kinase activity in transgenic mice expressing either human WT-SOD1 or G93A-SOD1, as well as in naïve, non-transgenic mice with only endogenous mouse SOD1. Spinal cord lysates were prepared from age-matched, presymptomatic (50d old) non-transgenic (naïve), WT-SOD1 (WT) and G93A-SOD1 (G93A) mice and processed for immunoblots (Fig. 4d). Phosphoantibodies against GSK3, JNK and ERK showed similar levels of activation for these kinases, regardless of genotype (not shown), but as seen previously [47]–[49], and in axoplasm (Fig. 4a), p38 phosphorylation increased in spinal cord lysates of G93A-SOD1, relative to those from WT-SOD1 and naïve mice. Interestingly, spinal cord lysates from WT-SOD1 mice showed a slight increase in p38 activation relative to naïve mice, suggesting a possible role for WT-SOD1 in modulating the p38 MAP kinase pathway or overexpression-related misfolding of WT-SOD1 [23]. SMI32 antibodies recognizing a dephosphorylated NF heavy chain (NFH) epitope showed reduced immunoreactivity in lysates from G93A-SOD1 relative to naïve and WT-SOD1 mice, indicating increased NFH phosphorylation at this site in G93A-SOD1 mice. Phosphorylation-dependent SMI31 antibodies mapping to a different NFH epitope showed similar immunoreactivity in naïve, WT-SOD1 and G93A-SOD1 mice, indicating that total NF levels and phosphorylation at the SMI31 epitope were unchanged. Anti-kinesin-1 (KHC) antibodies further confirmed similar protein loading. SMI32 antibodies recognize an NF220 epitope targeted by MAPKs, whereas SMI31 antibodies are not sensitive to MAPK activity [34]. Immunoblots of axoplasms and transgenic mice spinal cords, as well as co-perfusion experiments all indicated that pathogenic mSOD1 activate a p38 MAPK pathway. The increased ability of mSOD1, but not WT-SOD1 to increase axonal p38 kinase activity represents a toxic gain of function for mSOD1 consistent with autosomal dominant inheritance of mSOD1-related FALS.

### p38 MAPK activation in neurons of presymptomatic G93A-SOD1 transgenic mice

**Figure 5:**
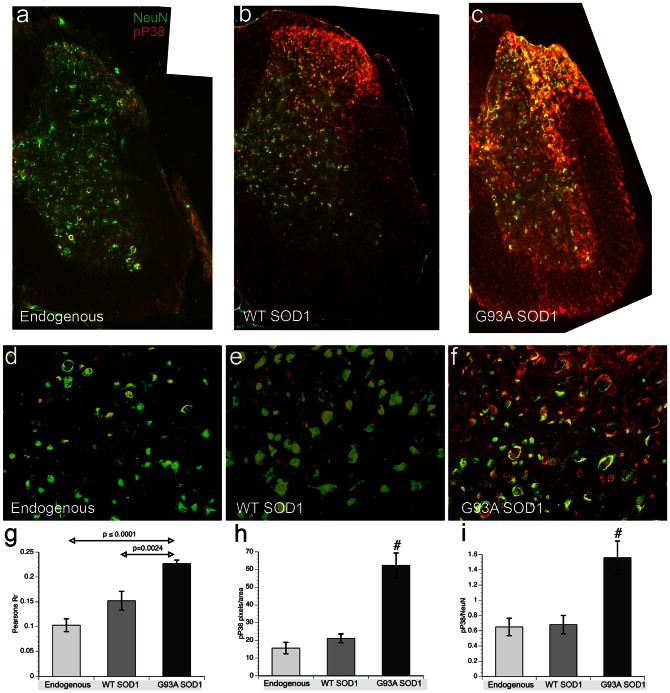
Increased Phospho-p38 in ventral horn of lumbar spinal cord in presymptomatic G93A-SOD1 mice. Spinal cords from 60 days old naïve nontransgenic mice (endogenous) and transgenic mice overexpressing either human WT- or G93A-SOD1 were analyzed by immunohistochemistry for cellular distribution of activated p38 MAPK. (**a–c)** Low magnification (10X objective) images of lumbar spinal cord double-labeled with a neuronal marker (NeuN, in green) and activated p38 MAPK (pP38, in red). **(d–f)** Higher magnification images (25× objective) of ventral horns. In naïve mice spinal cords with only endogenous mouse SOD1 (**a, d**), neurons are readily visible in the both dorsal and ventral horns (green), but p38 MAPK activity (red) is low with a few neurons positive for both markers (yellow), primarily in the dorsal horn. In transgenic WT-SOD1 spinal cord (**b, e**), neuronal staining is comparable to naïve mice, but p-p38 immunoreactivity clearly increases in regions with minimal NeuN staining, suggesting increased p38 MAPK activation in glia. In G93A-SOD1 spinal cord (**c, f**),p-p38 immunoreactivity is much higher in both white and grey matter regions,. An increase in pP38 co-localization with NeuN, is consistent with activation of p38 MAPK in neurons. **(g–i)** Semi-quantitative fluorescence analysis of high magnification sections shows an increase in co-localization of pP38 and NeuN immunoreactivity with G93A-SOD mice (n = 9 for all conditions). **(g)** Pearson's correlation coefficient (PCC) (showing co-localization, but not intensity levels) of NeuN and pP38 significantly increased in both transgenic mice relative to naïve mice, not quite rising to significance between naïve and WT-SOD1 (p = 0.058). In G93A-SOD1 mice, PPC values were significantly increased over both naïve and WT-SOD1 mice (p<0.0001). **(h)** To address relative levels of pP38 immunoreactivity, we compared red pixels/area. The difference between naïve and WT-SOD1 mice was not significant, but G93A-SOD1 mice was significantly different from both naïve and WT-SOD1 mice at p≤0.0001 (#) **(i)** An increased ratio of pP38/NeuN (R/G ratio) for G93A-SOD1 mice suggests that mutant SOD1 activates p38 in neurons. Differences between G93A-SOD1 and both naïve and WT-SOD1 mice were significant at p≤0.005 (#).

To determine whether p38 MAPK is activated within spinal cord motor neurons of mice expressing mSOD1, we compared age matched naïve mice to presymptomatic (60 days old) transgenic mice overexpressing either WT-SOD1 or G93A-SOD1 using antibodies recognizing p-p38 and NeuN, a neuron-specific marker (Fig. 5). Spinal cords from naïve mice (endogenous) showed low phospho-p38 immunoreactivity (Fig. 5a, d) consistent with low basal activation of p38 in normal tissue. Introduction of a WT-SOD1 transgene slightly increased phospho-p38 immunoreactivity (Figs. 5b, e and 4d), but there was minimal co-localization of phospho-p38 and NeuN in the ventral horn (Fig. 5g–i), with most p-p38 immunoreactivity mapping regions that lack neuronal cell bodies, such as white matter. In contrast, phospho-p38 increased significantly in both white and grey matter of G93A-SOD1 transgenic spinal cord (Fig. 5c, f, and 4d) and showed a significant increase in co-localization of p-p38 and NeuN indicative of neuronal activation (Fig. 5g-i). This is consistent with previous reports documenting activated p38 in motor neurons of mouse models of FALS, as well as in spinal cords from both SALS and FALS human patients [10], [47], [49]. Increased activation of p38 MAPK in neurons from 60 d old mice, before signs of motor neuron disease suggests an early step in FALS pathogenesis rather than an inflammatory response.

### Isoform-specific effects of p38 on FAT

**Figure 6:**
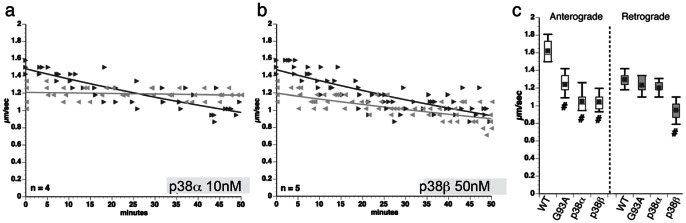
Active p38 α mimics the effects of pathogenic SOD1 on anterograde FAT. Effects of active, recombinant p38 isoforms on FAT were evaluated using vesicle motility assays in isolated squid axoplasm. P38α and P38β were perfused at a constant specific activity based on *in vitro* kinase assays with the ATF-2 substrate. **(a)** Perfusion of active p38α in axoplasm selectively inhibited anterograde FAT, as did pathogenic SOD1 (compare to Fig. 1b-d). (**b)** Unlike p38α, p38β inhibited both anterograde and retrograde FAT. (**c**) Quantitation of values obtained between 30-50 minutes shows that p38α most closely mimicked effects of pathogenic SOD1, suggesting this isoform mediates the effects of mSOD1 on FAT in axoplasm (#: difference significant from WT-SOD1 at p<0.01 by t-test).

Experiments in axoplasm suggested that pathogenic mSOD1 activate axonal p38 to inhibit anterograde FAT. but did not identify specific p38 isoforms. The complement of p38 isoforms expressed in squid is unknown, but four p38 genes exist in mammals (p38α, p38β, p38γ, and p38δ) [50], which differentially contribute stimulus- and cell-specific responses mediated by the p38 MAPK pathway. Of these, p38α and p38β are the major isoforms in the mammalian CNS, and in spinal motor neurons of the ventral horn, which are affected in ALS [51] (Fig. S2). Due to high homology conservation of the activation loop, anti p-p38 antibodies cannot distinguish between p38 isoforms, prompting us to evaluate functional effects of p38α and p38β on FAT in axoplasm (Fig. 6). Specific enzymatic activities of recombinant p38 isoforms were normalized using *in vitro* kinase assays with ATF-2 as substrate (not shown), allowing for perfusion of p38α and p38β at similar specific activities into axoplasm. As observed with G93A-SOD1, p38α (10 nM) selectively inhibited anterograde FAT (compare Figs. 1b and 6a). Effects of p38α on FAT were similar to G93A-SOD1 (Fig. 6c). In contrast, perfusion of p38β (50 nM) inhibited both anterograde and retrograde FAT (Fig. 6b–c). The relative selectivity of MW069 for p38α, and differential effects of p38α and p38β on FAT suggest that p38α may mediate the inhibition of FAT by mSOD1 in axoplasm. However, some upstream kinases for activation of p38α also activate p38β [52], so we cannot rule the possibility that mSOD1 activates both p38α and p38β in mammalian axons. Consistent with this possibility, sciatic nerve ligation experiments in G93A mice reported inhibition of both anterograde and retrograde FAT (data not shown) [14], [28], [53], [54].

### Conventional kinesin is a novel p38α substrate

**Figure 7:**
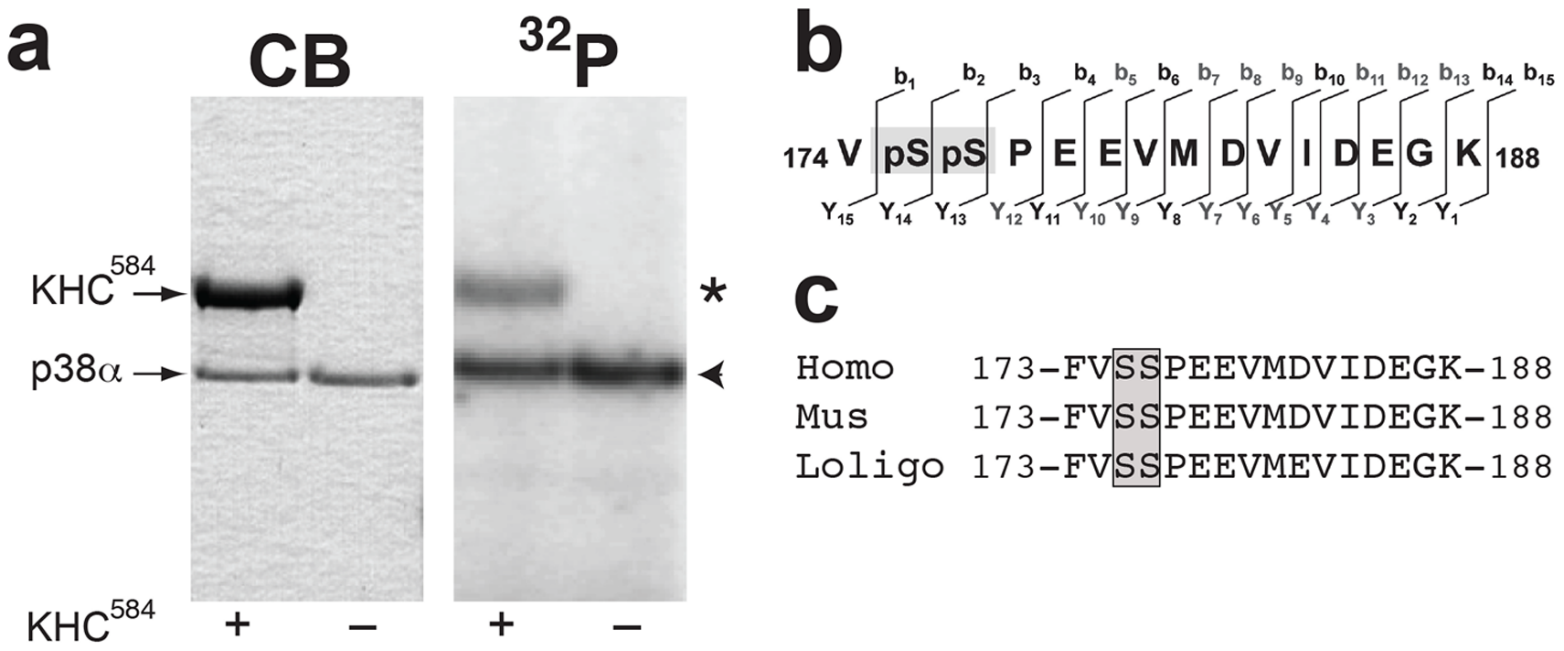
Active p38 α directly phosphorylates kinesin-1 at serines 175–176. **(a)** Recombinant p38α was incubated with γ-^32^P-ATP in the presence (+) or absence (–) of a recombinant protein construct comprising the first 584 amino acids of kinesin-1 (KHC^584^). Coomassie blue staining of gels shows the position of KHC^584^ and p38α. Autoradiogram (^32^P) shows ^32^P incorporation into KHC^584^ (asterisk), as well as autophosphorylation of p38α (arrowhead). **(b)** Mass spectrometry studies identified a peptide within the motor domain of kinesin-1 (amino acids 174–188) showing unequivocal evidence of phosphorylation by p38α. Tandem mass spectrometry analysis (MS/MS) by collision-induced dissociation further mapped phosphorylation on both Ser175 and Ser176 (grey box). **(c)** Sequence alignment shows that serines 175 and 176 (grey box) are conserved among human, mouse and squid sequences for kinesin-1.

To determine how p38 affected anterograde FAT we analyzed downstream targets of activated p38. Given that JNK3, a MAPK that is biochemically similar to p38α MAPK [55], phosphorylates kinesin-1, we tested phosphorylation of kinesin-1 by p38α. *In vitro* kinase assays showed that recombinant p38α phosphorylates both a recombinant kinesin-1 construct containing the first 584 amino acids of kinesin-1C (KHC^584^) (Fig. 7a), and native kinesin-1 immunoprecipitated from mouse brain (Fig. S3). A dually phosphorylated peptide corresponding to amino acids 174–188 in kinesin-1C was identified using liquid chromatography tandem mass spectrometry (LC/MS/MS). Tandem mass spectrometry analysis (MS/MS) by collision-induced dissociation further mapped phosphorylation sites to serines 175 and 176 (Fig. 7b and Fig. S4 and S5), consistent with the substrate preference of MAPKs for serines preceded by a proline, and with phosphorylation of Ser176 by JNK3. Sequence homology analysis showed Ser175 and 176 are conserved among squid, mouse and human kinesin-1s (Fig. 7c and Fig. S4 and S5). These experiments identified kinesin-1 as a novel p38α substrate, and mapped serines 175/176 as p38α acceptor residues.

### Ser175-176 phosphorylation inhibits kinesin-1 translocation

**Figure 8:**
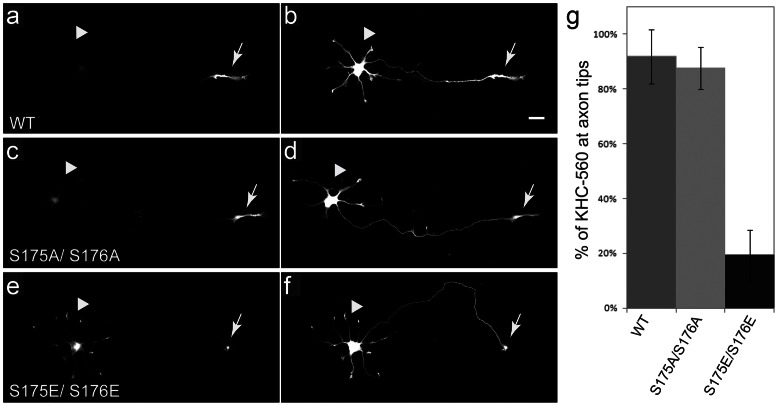
Pseudophosphorylation of kinesin-1 at S175/S176 inhibits movement of kinesin-1. To determine the effects of modifying S175 and S176 on kinesin-1function, recombinant GFP-tagged kinesin (KHC^559^) was modified to preclude phosphorylation at these sites (S175AS176A) or to mimic phosphorylation (S175ES176E). **(a–f)** Stage 3 hippocampal neurons were examined 5 h after co-transfection with GFP-tagged KHC^559^ constructs and a tdTomato construct, which diffuses throughout the cell and allows visualization of neurites (**b, d, f**). Both wild-type kinesin-1 (KHC^559^ WT, **a**) and a non-phosphorylatable mutant (KHC^559^ S175A/S176A, **c**) accumulated efficiently at axonal tips (labeled by arrows) with minimal steady-state labeling of cell bodies (arrowheads). In contrast, pseudophosphorylated mutant KHC^559^ S175E/S176E, **e**) was mainly present in neuronal cell bodies. Quantitative immunofluorescence analysis shows fraction of total KHC^559^ fluorescence at axon tips for all constructs **(g)**. Far less phosphomimicking KHC^559^ S175E/S176E constructs accumulated at axon tips than KHC^559^ WT or KHC^559^ S175A/S176A (#: p<0.001; *n*: 27–43 cells per condition). Bars show mean and standard deviation. Scale bar  = 20 µm.

Serines 175/176 are in a surface loop of the kinesin-1 motor domain implicated in binding to microtubules [56]. Phosphorylation of serine 176 by JNK3 reduces kinesin-1 binding to microtubules and inhibits kinesin-1 translocation along axonal microtubules *in vivo* [26]. To determine whether phosphorylation of kinesin-1 by p38 had a similar effect, GFP-tagged, kinesin-1 constructs were expressed in cultured hippocampal neurons to evaluate effects of dual S175/S176 phosphorylation on kinesin-1 motility *in vivo* (Fig. 8). These were co-expressed with soluble tdTomato to define axonal and dendritic processes [26] (Fig. 8b, d, f). A GFP-tagged kinesin-1 construct encompassing the first 559 amino acids of kinesin-1C (KHC^559^-GFP -WT) selectively translocates and accumulates at distal ends of axons, but not dendrites [26], [57]. Little or no fluorescence from KHC^559^-GFP-WT was detected in cell bodies or along axons, suggesting highly efficient translocation along axonal microtubules (Fig. 8a). We compared translocation of KHC^559^-GFP-WT (Fig. 8a) with that of the phosphomimetic construct KHC^559^-GFP-S175E/S176E (Fig. 8e), and the non-phosphorylatable control construct KHC^559^-GFP-S175A/S176A (Fig. 8c). Using quantitative fluorescence microscopy the amount of KHC^559^-GFP-S175A/S176A accumulated at axon tips did not differ significantly from KHC^559^- GFP-WT (88±8% vs. 92±10%, respectively, mean ± SEM) (Fig. 8g). In contrast, much lower levels of phosphomimetic KHC^559^-GFP-S175E/S176E construct accumulated at axonal tips, compared to KHC^559^-GFP-WT construct (19±9% vs. 92±10%, respectively, mean ± SEM; t-test, p<0.001). KHC^559^-GFP- S175E/S176E fluorescence was prominent in cell bodies with faint staining of neurites (Fig. 8e). Effects of double pseudophosphorylation were more dramatic than those with S175E single pseudophosphorylated KHC^559^ [26]. Thus, a mutation mimicking Ser175/176 phosphorylation dramatically reduces efficiency of kinesin-1 translocation along axonal microtubules in cultured neurons.

### SOD1 activates MAPKs upstream of p38

**Figure 9:**
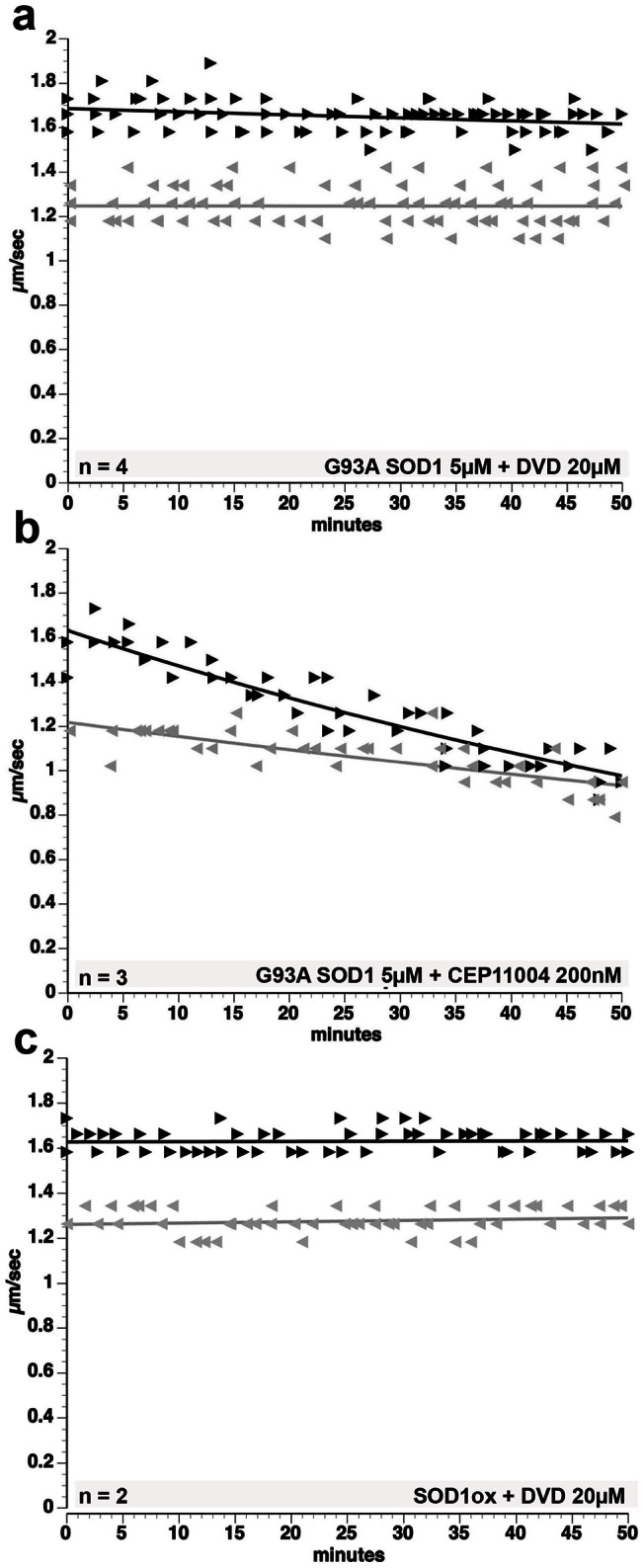
Inhibition of anterograde FAT induced by mSOD1 depends on specific MKKK-MKK interactions. Co-perfusion of G93A-SOD1 with DVD peptide **(a)**, but not with the Mixed-Lineage Kinase inhibitor CEP11004 **(b),** prevents inhibition of FAT induced by G93A-SOD1. DVD peptide prevents activation of MKKs by some MKKKs (n =  number of axoplasms) [60]. These data suggest that the activation of p38 and the inhibition of FAT induced by G93A-SOD1 involves activation of one or more MAPKKKs *other* than MLKs. **(c)** The DVD peptide also blocks inhibition of FAT by oxidized WT-SOD1 suggesting that FALS mutant SOD1 and misfolded WT-SOD1 activate a common p38 MAPK pathway [23].

To address mechanisms for activation of p38 by mSOD1, we looked at the role of upstream MAPK kinases. Activation of p38 involves phosphorylation by MAPK kinases (MKK3 and MKK6), which in turn are activated by MAPK kinase kinases (MKKKs) [58]. Members of several MKKK families can activate p38, including mixed-lineage kinases (MLKs), MEK kinases (MEKKs), apoptosis-inducing kinase (ASK1), TGFβ-activated kinase 1 (TAK1) and Thousand And One amino acids kinases (TAOs) [50], [58], [59]. Oxidized WT SOD1, SOD1 from SALS patients [23] and mSOD1 had similar effects on anterograde FAT and p38 MAPK activity, prompting us to ask whether they activate p38 MAPK by activating kinases upstream of p38. We co-perfused G93A-SOD1 or oxidized WT SOD1 with DVD peptide. Synthetic DVD peptides block p38 activation by inhibiting the docking and activation of MKKs by a subset of MKKKs, including MEKKs, ASK, TAO and TAK1 [60]. A DVD peptide containing a mutation that abolishes its blocking activity served as control [60]. Significantly, DVD peptide blocked effects of both G93A-SOD1 and oxidized WT-SOD1 on FAT (Fig. 9a,c), whereas control DVD peptide had no protective effect (Fig. S6). In contrast, pharmacological inhibition of mixed lineage kinases (MLK) by CEP11004 [61] failed to prevent G93A-SOD1 effects on FAT, suggesting that activation of axonal p38 by mSOD1 and oxidized SOD1 involves an MKKK upstream of p38 other than MLKs (Fig. 9b) [46].

## Discussion

Some 145 different mutations in *SOD1* are linked to FALS (http://alsod.iop.kcl.ac.uk/), accounting for roughly 20% of FALS cases. The characteristic pathological features of ALS are remarkably similar whether the disease is FALS or SALS, including axonal degeneration, abnormal patterns of phosphorylation, and FAT defects. In mSOD1-related FALS, pathogenic mutations are distributed throughout the molecule with diverse effects on enzymatic activity [62], suggesting that loss of SOD1 function is not a primary factor to mSOD1 pathogenesis. Analysis of the genetics and biochemistry of mSOD1 transgenic mice and human FALS indicated that pathogenic effects of FALS-linked mSOD1 in motor neurons represent a toxic gain of function [1], but the nature of this gained function was unclear.

An important step in defining pathogenic mechanisms for any disease is identification of pathognomonic features of the disease. Many ALS studies focus on mechanisms of motor neuron cell death, which occur through well-defined apoptotic pathways [63], [64]. However, death of motor neurons can be prevented without significantly altering the course of the disease [65], [66]. Early neurological symptoms of ALS relate better to loss of synaptic function and axonal connectivity than motor neuron cell death, which is a much later event in ALS [66]. However, both molecular events might be linked to abnormal activation of the p38 MAPK pathway by pathogenic SOD1 (Fig. S7).

Neurons affected in FALS exhibit a number of distinctive early changes, including abnormal activation of protein kinases, aberrant patterns of neurofilament phosphorylation, loss of synapses and impairments in FAT, among others [1]. However, distinguishing between primary pathognomonic events in ALS and secondary consequences of primary mechanisms has proven difficult [7]. In ALS, motor neurons typically degenerate following a “dying-back” pattern, characterized by altered synaptic function and axonal connectivity well before cell death [6], [65], [66]. Genetic data indicates that deficits in anterograde FAT *suffice* to produce dying-back degeneration of motor neurons [29], suggesting that FAT deficits might constitute a critical pathogenic event in ALS [7]. However, specific molecular components and mechanisms linking pathogenic mSOD1 to deficits in FAT were unknown.

Vesicle motility assays in isolated axoplasm allowed direct evaluation of mSOD1 effects on FAT independent of nuclear activity, transcription and glial-related effects. WT-SOD1 had no effect on FAT when perfused in axoplasm, but all FALS-linked SOD1 mutants tested (G93A, G85R and H46R) selectively inhibited anterograde, kinesin-based FAT (Fig. 1). Thus, inhibition of anterograde FAT represents a common toxic effect of both FALS-related mSOD1 (Fig. 1) and misfolded wild type SOD1 [23], [46]. G93A-SOD1 has wild-type-like SOD1 dismutase and metal binding activity, whereas G85R and H46R mutants are catalytically deficient, indicating that inhibition of FAT by FALS-linked mutants is unrelated to normal enzyme functions [67]. Selective inhibition of anterograde, but not retrograde FAT in axoplasm by pathogenic SOD1 proteins ruled out deleterious effects on microtubules, ATP availability, or general steric interference of motor proteins by mSOD1. Consistent with a prior report [28], no interaction was observed between SOD1 and kinesin in spinal cord from transgenic mice expressing WT-SOD1 or G93A-SOD1 (Fig. 1f).

Conventional kinesin and cytoplasmic dynein are regulated by phosphorylation of specific motor subunits. Abnormal activation of protein kinases and aberrant patterns of protein phosphorylation represent major ALS hallmarks [9] **.** Our previous studies in axoplasm suggested that pathogenic SOD1 might activate kinases involved in regulation of kinesin-based FAT [29]. Pharmacological, biochemical and immunochemical approaches indicated a role for p38 MAPK in inhibiting anterograde FAT (Figs. 3–7). Previous studies had demonstrated the presence of both p38α and p38β MAPK in neurons, with localization in both nuclei and cytoplasmic domains [68]. Activation of p38 MAPK has also been associated with axonal pathology [47], [69], but the p38 isoform was not determined and the axonal target of activated p38 MAPK was unknown [9]. Identification of kinesin-1 motor domains as a novel p38α kinase substrate provides a molecular basis for inhibitory effects of FALS-linked SOD1 mutants on anterograde FAT (Figs 8, and Fig. S8). Our findings were consistent with studies showing activation of p38 MAPK in motor neurons as an early pathogenic event in ALS [47], [51] and other reports showing that p38α may be enriched in neurons, whereas p38β was higher in microglia [70]. In mammals, expression of p38α (MAPK14) and p38β (MAPK11) is higher in ventral motor neurons of adult mouse spinal cord than in most cells of spinal cord and brain (Fig. S2), which may partially explain the unique vulnerability of motor neurons to FALS-linked mutants of SOD1, despite near ubiquitous expression of SOD1. In addition, transgenic mice expressing G93A-SOD1 exhibit significant activation of p38 MAPK in neuronal cells (Fig. 5). Curiously, p38 MAPK was slightly activated in spinal cords of mice overexpressing WT-SOD1, but this was primarily in white matter, presumably in glia and other non-neuronal cells.

The full complement of p38 isoforms in isolated axoplasm remains to be determined. Thus, selective inhibition of anterograde FAT by mSOD1 in squid axoplasm may result from either selective activation or selective expression of p38α In mammalian motor neurons, both p38 isoforms are expressed (Fig. 6) [68] and thus it is conceivable that mutant SOD1 may activate both α and β isoforms of p38 MAPK in mammals, ultimately affecting both anterograde and retrograde axonal transport. Regardless, the relative contributions of retrograde and anterograde FAT deficits to FALS pathogenesis in humans remains to be fully characterized.

In isolated axoplasms, kinesin-1 and cytoplasmic dynein-dependent FAT were evaluated directly by measuring the rate and amount of MBOs moving in both directions after challenge with FALS-linked mSOD1 (Figs. 1 and 3). Moreover, our studies identified differential isoform-specific effects of p38 on FAT, with p38α selectively inhibiting anterograde FAT and p38β affecting both directions (Fig. 6). Regardless of p38 isoforms involved, inhibition of FAT by SOD1 is likely to differentially affect specific kinesin-1 isoforms and MBO cargos. Different kinesin-1 isoforms move different MBOs [24], [71] and kinesin-1 isoforms may differ in sensitivity to p38 (unpublished data). A recent study found no correlation between changes in mitochondrial transport and axonal degeneration in mouse models of FALS [72], but, mitochondrial transport differs significantly from other FAT cargoes [73], [74], such as MBOs carrying synaptic vesicle precursors [35], [75], [76].

The selective effect of mSOD1 on anterograde, but not retrograde FAT in isolated axoplasm was unexpected, given reports describing inhibition of both anterograde and retrograde FAT in G93A-SOD1 mice [14], [28], [53], [54]. Some reports were based on reduced accumulation of FAT membrane proteins at a nerve ligation [14], [54]. Although informative, evaluation of retrograde FAT by this method could be affected by reductions in delivery of cargoes by anterograde FAT, and/or by reductions in synaptic activity leading to reduced commitment of material to retrograde FAT. Further, these experiments were performed in mice that were 85 days [54] and 133 days old [14], ages at which major denervation has occurred [6]. Reduced delivery of retrogradely transported cargoes has important implications for neuronal survival, but nerve ligation experiments do not directly evaluate changes in motor protein function.

Suggestions of a role for retrograde FAT deficits in prior FALS studies [54], [77]–[79] also stem from genetic studies reporting motor neuron degeneration in mice with some cytoplasmic dynein heavy chain mutations (i.e. *Legs at odd angles (Loa)*, and *Cramping 1 (Cra1)*, but not *Sprawling* [80]). Subsequent studies showed that Loa and Cra1 mutants exhibit severe proprioceptive sensory neuron loss before any loss of motor neurons [81], raising concerns on the use of these mice as models of motor neuron disease [82]. Curiously, some dynein mutations extended FALS mutant transgenic mouse lifespan [83], [84], while other mutations did not [85]. Collectively, genetic evidence demonstrated that specific perturbations in cytoplasmic dynein-dependent retrograde FAT may result in peripheral sensory neuropathy, but inhibition of retrograde FAT alone might not be sufficient to produce motor neuron disease [86].

In summary, FALS-linked mSOD1 activates a p38 MAPK pathway in squid axoplasm and mammalian spinal cord, which in turn phosphorylates kinesin-1 motor domains leading to reduced ability of kinesin-1 to move along axonal microtubules (Fig. 8 and Fig. S8). Disruption of FAT leading to loss of synaptic function and dying–back axonopathy establishes mSOD1-linked FALS as a dysferopathy, i.e. a pathology associated with compromised fast axonal transport leading to a late-onset dying back neuropathy [29], [87], resulting in this case from aberrant signaling through the p38 pathway. Clearly, p38 MAPK has substrates other than kinesin-1, including neurofilaments and proteins involved in transcription or proapoptotic pathways (Fig. S8). Alterations in the phosphorylation of multiple p38 targets may contribute in significant ways to ALS pathology [88]. Therefore, targeting signaling pathways between mSOD1 and p38 represent a promising new direction for therapeutic intervention in ALS, particularly since this pathway is also implicated in some SALS cases [23], and brain-permeable p38 kinase inhibitors exist [45].

## Materials and Methods

### Ethics Statement

All animal work was done according to guidelines established by the NIH and the corresponding institutions and are covered by appropriate institutional animal care and use committee protocols from the University of Illinois at Chicago Animal Care Committee (approval #11–201, and #11–180) and Oregon Health Sciences University Institutional Animal Care and Use Committee (approval #A607)). There were no primates or human subjects involved in any of these studies, so these experiments are not eligible for consideration by Institutional Review Boards for protection of human subjects. The animal care and use committees at UIC and OHSU are responsible for evaluating all ethical and welfare issues regarding vertebrate animals. No vertebrate animals or human embryonic cell lines were used at other sites. All studies were conducted at institutions in the USA.

### Antibodies and Reagents

The following antibodies were used: anti-KHC (H2 clone) [24], anti-SOD1 (D3H5 clone, a generous gift from Dr. Jean-Pierre Julien), SMI31 and SMI32 antibodies (Sternberger), anti-phospho JNK (Cell Signaling #9252), anti-phospho p38 (Cell Signaling #9215), anti-phospho GSK3 (Santa Cruz #11757), anti-phospho ERK (Santa Cruz #7383). The secondary antibodies used were Jackson 111–035–045 HRP-conjugated goat anti-rabbit, Jackson 115–035–146 HRP-conjugated goat anti-mouse IgG, and Jackson 805–035–180 HRP-conjugated bovine anti-goat IgG. Primary antibodies for immunohistochemistry were anti phospho p-38 MAPK (pP38) (Cell Signaling cat#4511) and anti- NeuN (Novus Biological cat# NBP1-9269). Secondary antibodies were goat anti-rabbit Alexa 594 (Invitrogen cat# A11012) and anti-mouse Alexa 488 (Invitrogen cat# A11011). Cyclosporine A, SB203580 and SP600125 (JNK inhibitor II) were obtained from Calbiochem. CEP11004 was a gift from Cephalon. MW01-6-189WH (MW189) and MW01-2-069SRM (MW069) are described in [45]. His-tagged KHC^584^ protein constructs were expressed in BL21-Codon Plus (DE3) *E. coli* (Stratagene), and purified using Talon beads (Clontech), as before [26]. Recombinant p38 isoforms were from Upstate (p38α #14–251, p38β #14–253). Recombinant SOD1 constructs were prepared in insect cells as before [21].

### Vesicle motility assays in isolated axoplasm

Axoplasms were extruded from giant axons of the squid *Loligo pealii* (Marine Biological Laboratory) as described previously [19]. Recombinant proteins, peptides or inhibitors were diluted into X/2 buffer (175 mM potassium aspartate, 65 mM taurine, 35 mM betaine, 25 mM glycine, 10 mM HEPES, 6.5 mM MgCl_2_, 5 mM EGTA, 1.5 mM CaCl_2_, 0.5 mM glucose, pH 7.2) supplemented with 2–5 mM ATP (Figs. 1, 3, 6) and 20 µl added to perfusion chambers. For biochemical experiments in isolated axoplasm, a mixture of 1mM ATP and 0.1 mCi ^32^P-labelled ATP was used. For vesicle motility assays, preparations were analyzed on a Zeiss Axiomat with a 100×, 1.3 n.a. objective, and DIC optics. Hamamatsu Argus 20 and Model 2400 CCD camera were used for image processing and analysis. Organelle velocities were measured with a Photonics Microscopy C2117 video manipulator (Hamamatsu) as described previously [38]. For quantitative comparisons between conditions, velocity measurements taken 30–50 minutes after perfusion were pooled and the mean velocities calculated. Rates obtained by this method reflect a sampling of vesicle movements in and out of the plane of focus. Therefore, recorded velocities correlate with *both* rate and number of vesicles moving in a given treatment (i.e. low transport rates may reflect reduced number of vesicles moving, as well as slower velocities [19], [38], [89]}.

### Immunochemical methods

Spinal cords from transgenic mice expressing WT-SOD1 and G93A-SOD1 were homogenized in lysis buffer (LB; 25 mM Tris pH 7.4, 150 mM NaCl, 1% Triton X-100, and mammalian protease inhibitor cocktail (Sigma, 1/100 dilution) as described previously [24]. Quantitative immunoblots were performed as described previously [26] using SDS-PAGE on 4–12% Bis-Tris gels (NuPage minigels, Invitrogen), using MOPS Running Buffer (Invitrogen) and transferred to PVDF using Towbin buffer supplemented with 10% (v/v) methanol. Orthovanadate (1 mM) and Sodium Fluoride (10 mM) were in all incubation steps involving phosphoantibodies. Primary antibody binding was detected with HRP-conjugated anti-mouse, anti-rabbit or anti-goat antibodies (Jackson Immunoresearch), and visualized by chemiluminescence (ECL, Amersham).

### Immunohistochemistry

Experiments followed approved Institutional animal protocols at the University of Illinois in Chicago. Sixty day old transgenic WT-SOD1 (JAX#002297, 3 Female), G93A-SOD1 (JAX #002726, 2 female and 1 male) mice, and nontransgenic littermates (3 male) were euthanized by carbon monoxide inhalation, and transcardially perfused with PBS and 4% solution of paraformaldehyde (PFA) in PBS. Tissues were process for sectioning and embedded in OCT (Tissue Tek, cat #4583) 50 µm thick spinal cord sections were obtained and mounted on slides. OCT was removed and sections permeabilized with Triton-X100. Primary antibodies were anti phospho p-38 MAPK (pP38) (1∶200) and anti- NeuN (1∶400). Secondary antibodies were goat anti-rabbit Alexa 594 and anti-mouse Alexa 488 used at 1∶1000 dilution. Slides were dried and mounted in VectaShield mounting media (Vector Laboratories, Burlingame, CA) and sealed with nail varnish.

Spinal cord images such as those in Fig 5 were assembled from images obtained with a 10× objective. For quantitation of pP38 immunoreactivity (Fig. 5 h–i), images of the spinal cord ventral horn were obtained using a 25X objective (n = 3 animals per group). Red and green channels (phospho-p38 MAPK and NeuN, respectively) were transformed to 8 bit images using ImageJ software (http://imagej.nih.gov/ij/) and mean pixel values per equal area on each image quantified by auto-threshold methods (Fig. 5h). In order to determinate relative pP38 levels in NeuN-positive cells, an index; pP38/NeuN was generated (Fig. 5i). Co-localization index values represented by the Pearson's correlation values were obtained using the co-localization finder toolbox in ImageJ. [90] (Fig. 5g).

### In vitro Phosphorylation

*In vitro* phosphorylation experiments (40 µl volume) were performed by incubating KHC^584^ protein constructs (3 µM) with 0.1?M p38α in HEM buffer (50 mM HEPES, 12 mM MgSO4, 100 µM ATP pH 7.4), as previously described [26].

### Mass spectrometry studies

Phosphorylated KHC584 protein was subjected to in solution trypsin digestion for LC/MS/MS analysis as described previously [26]. Briefly, dried samples were resuspended in 30 mM HEPES and 30 mM NaF in the presence of 1 µg of trypsin (Sigma, proteomics grade) and incubated at 37°C overnight (16–18 h). The resulting peptides were later resuspended in buffer A (5% acetonitrile, 0.4% acetic acid, 0.005% heptafluorobutyric acid (vol/vol) in water) for mass spectrometry analysis. Samples were analyzed by high-pressure liquid chromatography (HPLC, 11-cm by 100-µm fused silica capillary column packed with reverse C18 material, Magic beads, Michrom Bioresources) on line with a LTQ (a two-dimensional ion trap) instrument equipped with a commercial nano-electrospray source (Thermo Finnigan). Samples were automatically loaded by a microautosampler (Famos, LC Packings). Samples were analyzed by performing full scan followed by tandem mass spectrometry or MS/MS of the five most intense ions (top five) by collision-induced dissociation. Sample loading, solvent delivery and scan function were controlled with Xcalibur software (Thermo Finnigan). LC/MS/MS files were searched using a SEQUEST algorithm against a database containing KIF5C_Rat and KIF5C_Mouse, among others proteins. SEQUEST search parameters included mass tolerance of ±1.5 Da, and a differential search for serine, threonine and tyrosine phosphorylation. The dataset was filtered using INTERACT based on the following criteria: delta correlation of 0.1, and X correlation values for +1 peptides ≥1.8,+2≥2.15 and +3≥3.2.

### Kinesin-1 Translocation Assays

S175A/ S176A and S175E/ S176E mutant variants of rat GFP-tagged KIF5C^559^ constructs were prepared by site-directed mutagenesis. Experiments evaluating the accumulation of these constructs were performed as before [57]. Primary hippocampal cultures with glial feeder layers were prepared from E18 embryonic rats. After 2 days in culture, hippocampal neurons were co-transfected with plasmids pBA-KIF5C559^WT^-GFP (Jacobson et al., 2006), pBA-KIF5C559^S175A/176A^-GFP, or pBA-KIF5C559^S175E/176E^-GFP together with soluble tdTomato protein using Lipofectamine 2000 (Invitrogen, Carlsbad, California). Five hours after transfection, cells were fixed with 4% paraformaldehyde. Fluorescence images were taken using a Zeiss Axio Observer Z1 microscope (Carl Zeiss, Thornwood, NY). To determine fraction of KIF5C^559^ signal at axon tips, the integrated fluorescence intensity of the whole cell and the distal axon were calculated after subtracting background using Metamorph software (Molecular Devices Corporation, Downingtown, PA).

### Statistical Analysis

All experiments were repeated at least 3 times. Unless otherwise stated, the data was typically analyzed by pooled t-test of µ1–µ2 using DataDesk statistical software. Quantitative data was expressed as mean ± SEM unless otherwise stated and significance was determined at *p*<0.05 or 0.01 as noted. P values are given as calculated.

## Supporting Information

Text S1 **Supplementary Results and Methods.** Supplemental results are provided showing the expression of p38 MAP kinase α and β in the adult mouse spinal cord, indicating that these kinases are highly expressed in ventral motor neurons. Results of experiments showing that activation of p38 MAP kinases by mutant SOD1 compromises cell viability as well as axonal transport, providing a link between this pathway and the loss of motor neurons in SOD1-related ALS. An expanded description of the methods used in this study is provided to facilitate future studies.
(DOCX)

Figure S1 **Outline of metabolic labeling experiments and immunobloting analysis in isolated squid axoplasm.** Two giant axons were dissected from the same squid (“sister” axons), extruded, placed on glass coverslips, and incubated with recombinant SOD1 proteins. One axon was perfused with WT-SOD1, whereas the contralateral axon was perfused with pathogenic SOD1. For metabolic labeling experiments in Figure 2, an aliquot of radiolabelled ^32^P-ATP was added to each axoplasm. After a 50-minute incubation, axons were lysed and processed for autoradiography (Fig. 2) or immunoblotting (Fig. 4).
(TIF)

Figure S2 **Expression of p38**α**/MAPK14 and p38**β**/MAPK11 in spinal cord is enriched in ventral motor neurons.** Data from the Allen Mouse Spinal Cord Atlas (http://mousespinal.brain-map.org/) shows expression of p38 MAPK α and β isoforms of the lumbar spinal cord of an adult mouse. Based on location, Nissl staining and somal size, the large cells in the ventral horn are identified as alpha motor neuron cell bodies (see arrowheads for examples). The panels are **(a)** *in situ* hybridization of a section of lumbar spinal cord from an adult mouse showing that distribution of p38α/MAPK14 mRNA is enriched in the cytoplasm of large neurons, particularly in motor neurons of the ventral horn; and **(b)** expression mask derived from the in situ data shows differential expression of the target gene (p38α/MAPK14) with black reflecting no detectable expression, blue showing low expression with green and yellow representing increasing levels of expression. **(c)** *In situ* hybridization of a section of lumbar spinal cord in an adult mouse showing that p38β/MAPK11 mRNA also exhibits higher expression in large cells in the ventral horn, presumptive alpha motor neurons; and **(d)** expression mask showing differential expression of the target gene (p38β/MAPK1). Both p38α and p38β are preferentially expressed in motor neurons.
(TIF)

Figure S3 **Recombinant p38**α **directly phosphorylated both recombinant KHC and immunoprecipitated endogenous brain KHC. (a),** Recombinant p38α was incubated in the presence (+) or absence (–) of KHC (KHC584) recombinant protein. An autoradiogram shows incorporation of ^32^P into KHC584 (*) and autophosphorylated JNK. **(b)**, Recombinant p38α was incubated with immunoprecipitated, endogenous mouse brain kinesin-1. The autoradiogram (^32^P) shows increased phosphorylation of KHC. The accompanying western blot (WB) shows equal amounts of immunoprecipitated KHC in each condition.
(TIF)

Figure S4 **Mass spectrometry analysis of kinesin-1 phosphorylation by p38α.** (**a**) Diagram of mass spectrometry procedures for the analysis of kinesin-1 phosphorylation by p38α showing the path that a protein sample follows during High Performance Liquid Chromatography Mass spectrometry analysis (HPLC-MS. Peptides generated by trypsin treatment of samples are first resolved by a reversed phase column. After peptides elute from the column, ions for mass spectrometry analysis are generated by Electrospray Ionization (ESI). Once peptides enter the mass spectrometer, the most abundant ions are individually selected and captured to go under Collision Induced Dissociation (CID), which yields a collection of shorter sequences for peptide identification. The output of each individual peptide analysis is a mass spectrum that is analyzed by bioinformatics to match to a known protein in the database for protein identification. (**b**) Actual mass spectrum of the KIF5c 174–188 phosphopeptide. The graph shows the output mass spectrum, obtained from the mass spectrometer, for one of the identified peptides of the KIF5c protein. The graph plots ion intensity versus mass to charge ion ratio (M/Z) for ***b+*** (red) and ***y+*** (blue) ions that are the direct (N to C terminus) and reverse (C to N terminus) ion series obtained during CID. The identified amino acids peptide sequence for this spectrum is shown in the upper right of the spectrum.
(TIF)

Figure S5 **p38α phosphorylation sites on kinesin-1.** **(a)** The table shows the identified phosphopeptides in the KIF5c rat sequence from recombinant KIF5c phosphorylated by p38α in vitro. From left to right, the table shows the protein ID entry for the database utilized in protein identification analysis; the sequence of the identified phosphopeptide; the mass to ion charge ratio that corresponds unequivocally to that ion or peptide; peptide position in the sequences of the protein (KIF5c) given by the position of the amino (N terminus) and carboxyl (C terminus) amino acid residue; and the last column indicates the position of the actual phosphorylated residue. Of these peptides, the only sites conserved between human, mouse and squid kinesin-1 protein were S175/S176 in peptide 173–190 (shown in red). **(b)** Several parameters are shown for the identified phosphopeptides. From left to right: File name of the mass spectrum obtained from the mass spectrometer for the peptide, total mass of the ion or peptide, x correlation (XCorr) and delta correlation value (dCn) for each identified peptide. These two parameters emerge from the bioinformatic data analysis after mass spectrometry. These values are used to decide whether a peptide should be reported or not. The cut off values were specified in materials and methods (see above). The next two columns indicate the number of identified peptide during CID and the total number of theoretical ions. Finally, the protein name entry in the database and the peptide sequence are given.
(TIF)

Figure S6 **DVD Peptide Prevents Inhibition of FAT by mutant SOD1.** Co-perfusion of G93A-SOD1 with DVD peptide **(a)**, but not with a control DVD peptide **(b)** prevents inhibition of FAT induced by G93A-SOD1. DVD peptide prevents activation of MKKs by some MKKKs (n =  number of axoplasms), whereas DVD control peptide does not. These data suggest that the activation of p38 and the inhibition of FAT induced by G93A-SOD1 involve activation of one or more MAPKKKs that require the DVD docking motif for activation of downstream kinases.
(TIF)

Figure S7 **Inhibition of p38 attenuates mSOD1-induced apoptosis.** Expression of mutant SOD1 in N2A cells has a very modest effect on cell viability that is greatly enhanced by challenge with cyclosporine A (CsA). Stably transfected N2A cells expressing WT-SOD1 (WT-SOD1, left panels) or G85R-SOD1 (mSOD1, right panels) were incubated with various concentrations of CsA, (0 to 14 µg/ml) in the presence (+) or absence (–) of the p38 inhibitor SB203580 (10 µM). **(a)** LDH toxicity assays show a dose-dependent increase in CsA-induced cytotoxicity on both WT-SOD1 and mSOD1 cell lines. However, the toxic effect of CsA is more pronounced in cells expressing mSOD1 (hatched red bars), compared to cells expressing WT-SOD1 (black striped bars). Remarkably, treatment of mSOD1 N2A cells with the p38 inhibitor SB203580 significantly attenuated cell death at 0, 3.5 and 7 µg/ml CsA (solid bars). In contrast, SB203580 reduced CsA-induced toxicity at 7 µg/ml, but not 3.5 µg/ml (solid bars) in WT-SOD1 N2A cells. Data represent the mean ± SEM % cytotoxicity for n = 8 wells (* p<0.0001). **(b)** Caspase-Glo assays confirmed and extended results in **a**, showing that CsA induced the activation of the pro-apoptotic caspases 3 and 7 in mSOD1 N2A cells (red hatched bars) to a greater extent than WT-SOD1 N2A cells (black striped bars). Treatment of mSOD1 N2A cells with SB203580 (solid bars) significantly attenuated caspase 3/7 activation, whereas WT-SOD1 cells exhibited similar caspase activity levels in the presence (solid bars) and absence (striped black bars) of SB203580. Data represent the mean ± SEM luminescence signal for treated cells relative to untreated cells for n = 4 wells (* p<0.0001). These results suggest p38 activity contributes to the increased vulnerability of mSOD1 N2A cells to CsA-induced cell death.
(TIF)

Figure S8 **Inhibition of conventional kinesin-based motility induced by pathogenic SOD1.** Our results showing increased activation and phosphorylation of p38 by mSOD1 polypeptides suggest that these pathogenic mSOD1 polypeptides activate specific MAPKKKs and MAPKKs (dashed arrow) upstream of p38 (Fig. 9). Activation of axonal p38 would lead to phosphorylation of kinesin-1, neurofilaments (NFs) and likely other axonal substrates. Data in this work indicates that phosphorylation of kinesin-1 by p38 inhibits translocation of conventional kinesin along microtubules. Reductions in the delivery of critical axonal cargoes by conventional kinesin, (such as synaptic vesicle precursors and organelles containing neurotrophin receptors) would result in impaired synaptic function and dying-back degeneration of neurons In addition, increased p38 activation in neuronal cell bodies would be expected to promote alterations in the activity of various transcription factors (i.e., ATF-2 and c-Jun, among others), consistent with reports of transcriptional changes and activation of apoptosis induced by pathogenic SOD1 expression.
(TIF)
