# Supplementary material for: Inhibition of Fast Axonal Transport by Pathogenic SOD1 Involves Activation of p38 MAP Kinase
Source: PLoS One. 2013 Jun 12;8(6):e65235. doi: 10.1371/journal.pone.0065235 (PMC3680447; doi:10.1371/journal.pone.0065235)
Supplement: Text S1 — Supplementary Results and Methods. Supplemental results are provided showing the expression of p38 MAP kinase α and β in the adult mouse spinal cord, indicating that these kinases are highly expressed in ventral motor neurons. Results of experiments showing that activation of p38 MAP kinases by mutant SOD1 compromises cell viability as well as axonal transport, providing a link between this pathway and the loss of motor neurons in SOD1-related ALS. An expanded description of the methods used in this study is provided to facilitate future studies. (DOCX) [file pone.0065235.s001.docx]

**SUPPLEMENTAL RESULTS**

***Allen Brain Atlas Data on Expression of p38 MAPK isoforms.***

If activation of p38 MAPK by pathogenic forms of SOD1 is related to the disease process in ALS, then p38 MAPK isoforms should be expressed at significant levels in upper and lower motor neurons. Data from the Allen Mouse Spinal Cord Atlas (<http://mousespinal.brain-map.org/>) [[1](#_ENREF_1)] indicates that both p38 MAPK α and β isoforms are expressed in neuronal-rich regions of the lumbar spinal cord of an adult mouse (Fig. S2). *In situ* hybridization shows the highest levels of expression in this region localized to large cells of the ventral horn, presumptive alpha motor neurons. These data indicate that α and β isoforms of p38 MAPK are expressed in cells vulnerable in ALS

***p38 activation by mSOD1 affects neuronal cell viability.***

Multiple studies documented activation of apoptosis in association with mSOD1 expression [[2](#_ENREF_2),[3](#_ENREF_3)]. Most experiments in this study focused on axon-autonomous effects of mSOD1, but p38 kinase activation can be proapoptotic in some situations [[4](#_ENREF_4)]. Thus, the possibility existed that mSOD1-induced increase in p38 activity revealed in these studies may be relevant not only to the axonal compartment, but also to neuronal viability [[2](#_ENREF_2),[5](#_ENREF_5)]. Previous studies with N2A neuron-like cells showed that mSOD1 expression selectively increased apoptotic cell death induced by low levels (≤10μM) of cyclosporine A (CsA), whereas WT-SOD1 expression did not increase vulnerability to challenge with CsA [[5](#_ENREF_5)]. CsA-induced cell death assays provide a useful method for assessing the efficacy of various compounds in blocking or reducing mSOD1-mediated cytotoxicity. To evaluate the role of p38 on mSOD1-induced apoptosis in this model, we performed cell toxicity (LDH) and apoptosis activation (Caspase-Glo 3/7) assays (Fig. S7) using N2A cells stably transfected with either WT-SOD1 or G85R-SOD1 constructs. N2A cells were exposed to increasing concentrations of CsA in the presence or absence of the p38 inhibitor SB203580. Significantly, results from both LDH (Fig. S7a) and caspase-3 activity assays (Fig. S7b) showed SB203580 selectively attenuated CsA-induced cell death (Fig. S7a) and caspase-3 activation (Fig. S7b) in G85R-SOD1-expressing cells. Further, even in the absence of CsA, SB203580 prevented the modest increase in cell death induced by G85R-SOD1 expression alone. In contrast, these parameters were largely unchanged by SB203580 in WT-SOD1-expressing cells, except for a modest improvement of cell viability at 7µg/ml CsA. Taken together, these results suggest that activation of p38 contributes to mSOD1-mediated apoptosis.

**SUPPLEMENTAL METHODS**

***Kinesin-1 Immunoprecipitation***

Spinal cords from transgenic mice expressing WT-SOD1 and G93A-SOD1 were homogenized in lysis buffer (LB; 25 mM Tris pH 7.4, 150 mM NaCl, 1% Triton X-100, and mammalian protease inhibitor cocktail (Sigma, 1/100 dilution)]. Lysates were centrifuged twice for 5 minutes at 55,000 rpm (163,640 g_max_) using a TLA 100.3 rotor (Beckman Instruments, Palo Alto, CA). The resulting supernatant fractions were precleared using a mixture of Protein G agarose beads (Pierce), and non-immune mouse IgG-conjugated Sepharose beads (Jackson Immunoresearch) for 1 hour at RT. 400 µg of each precleared brain lysate were brought to 1 ml with LB, and incubated with 5µg of H2 antibody plus 10µl of Protein G agarose beads at 4^0^ C for 3hs [[6](#_ENREF_6)]. Immunocomplexes were recovered by centrifugation (3000 g_max_ for 30 seconds), washed four times with 1 ml LB, once with 50mM HEPES pH 7.4, and resuspended in Laemmli buffer.

***Immunoblots.***

Proteins were separated by SDS-PAGE on 4-12% Bis-Tris gels (NuPage minigels, Invitrogen), using MOPS Running Buffer (Invitrogen) and transferred to PVDF using Towbin buffer supplemented with 10% (v/v) methanol (90 minutes at 400mA using Hoeffer TE22 apparatus). Immunoblots were blocked with 1% (w/v) non-fat dried milk diluted in TBST (25 mM Tris pH 7.2, 2.68 mM KCl, 136.8 mM NaCl, 0,01% Tween-20, pH 7.2). Sodium Orthovanadate (1 mM) and Sodium Fluoride (10 mM) was used in all incubation steps involving the use of phosphoantibodies, after correcting for pH. Membranes were incubated with primary antibodies overnight at 4º C in 1% IgG free-BSA (Jackson Immunoresearch), and washed four times with 0.1% Tween-20 in TBS. Primary antibody binding was detected with HRP-conjugated anti-mouse, anti-rabbit or anti-goat antibodies (Jackson Immunoresearch), and visualized by chemiluminescence (ECL, Amersham). Quantitative immunoblotting was performed as before [[7](#_ENREF_7)].

***Immunohistochemistry***.

Experiments were performed following approved Institutional animal protocols at the University of Illinois in Chicago. Sixty day old transgenic WT-SOD1 (JAX#002297, 3 Female), G93A-SOD1 (JAX #002726, 2 female and 1 male) mice, and nontransgenic littermates (3 male) were euthanized by carbon monoxide inhalation, and transcardially perfused with PBS and 4% solution of paraformaldehyde (PFA) in PBS. Tissues were kept overnight in PFA and incubated in progressively increasing sucrose solutions (10-30%) for additional 24h. After embedding in OCT solution (Tissue Tek, cat #4583) 50µm thick spinal cord sections were obtained using a microtome (Leica CM 1850 Cryostat, Buffalo groove, IL). Sections were mounted on slides (Fisher brand Superfrost, cat# 12-550-15) and dried out for 15 minutes. The OCT was removed by washing three times with Tris base buffer (TBS). Sections were permeabilized with Triton-X100 0.25% for 10 minutes and blocked with 5% goat serum in TBS.. The primaries antibodies include anti phospho p-38 MAPK (pP38) (Cell Signaling cat#4511, 1:200] and anti- NeuN Novus Biological cat# NBP1-92693; 1:400). Secondary antibodies were goat anti-rabbit Alexa 594 (Invitrogen cat# A11012) and anti-mouse Alexa 488 (Invitrogen cat# A11011), both used at 1:1000 dilution. Slides were dried and mounted in VectaShield mounting media (Vector Laboratories, Burlingame, CA) and sealed with nail varnish.

Spinal cord images such as those in Fig. 5A-C were assembled from images obtained with a 10X objective. For quantitation of pP38 immunoreactivity (Fig. 5 H-I), three images were obtained from the ventral horn of the spinal cord using a 25X objective from each animal analyzed (n=3 animals per experimental group). Background subtraction was performed using samples without primary antibody. Images from red and green channels (phospho-p38 MAPK and NeuN, respectively) were transformed to 8 bit images using ImageJ software (<http://imagej.nih.gov/ij/>) and mean pixel values per equal area on each image quantified by auto-threshold methods (Fig. 5h). In order to determinate relative pP38 levels in NeuN-positive cells, an index; pP38/NeuN was generated (Fig. 5i). Further co-localization index values represented by the Pearson’s correlation values were obtained using the co-localization finder toolbox in ImageJ (<http://rsb.info.nih.gov/ij/plugins/colocalization-finder.html>). Specifically, imaging data was gathered in two independent channels: 594nm for phospho-P38 and 488nm for NeuN. Normalized pixel grey values were displayed in a pixel distribution scattered plot diagram (Fluorogram). The spread of distributions were used to estimate the goodness of fit in a linear range between both fluorophores and, as the slope reflects the relative stoichiometry of both fluorochromes, we can estimate a correlation coefficient on the fitted line from every set of images [[8](#_ENREF_8)] (Fig. 5g).

***Spinal Cord/axoplasm lysates.***

Spinal cords from either sex and axoplasms were homogenized in 1% SDS in 50mM Hepes pH7.4. Lysates were clarified by centrifugation, and protein concentration determined using BCA kit (Pierce) when needed.

***LDH and Caspase activity assays.***

Mouse neuroblastoma Neuro2A (N2A) cell lines stably transfected to express either WT-SOD1 or G85R-SOD1 in the Brown laboratory [[3](#_ENREF_3)] were maintained as described [[2](#_ENREF_2)]. Cells were plated in 96-well plates at a cell density of 6.0e^4^ cells/ mL and 2.35e^5^ cells/ mL, respectively, for the CytoTox 96 cytotoxicity assay (Promega), which measure lactate dehydrogenase (LDH) activity, and Caspase-Glo 3/7 (Promega) assay. Both assay were performed and analyzed according to the manufacturer’s instructions. Cells (n=4-8 wells per condition) were treated overnight (≈12 h) with 10 μM SB 203580 at 37°C and 5% humidity, and then treated with fresh media containing 0, 3.5, 7 and 14 µg/mL cyclosporine A (CsA; Calbiochem) for 24 hours [[2](#_ENREF_2),[5](#_ENREF_5)]. Statistics were performed on replicate wells (n=4-8) to assess the significance of p38 MAPK inhibition for each cell line at each CsA concentration (unpaired, two-tailed, t-test using Graphpad Prisms software). Results are representative of at least 3 experiments for each assay.

**SUPPLEMENTAL FIGURES.**

**Expanded legends.**

**Figure S1.** **Outline of metabolic labeling experiments and immunobloting analysis in isolated squid axoplasm.**

Two giant axons were dissected from the same squid (“sister” axons), extruded, placed on glass coverslips, and incubated with recombinant SOD1 proteins. One axon was perfused with WT-SOD1, whereas the contralateral axon was perfused with pathogenic SOD1. For metabolic labeling experiments in Figure 2, an aliquot of radiolabelled ^32^P-ATP was added to each axoplasm. After a 50-minute incubation, axons were lysed and processed for autoradiography (Fig. 2) or immunoblotting (Fig. 4).

**Figure S2. Expression of p38**α**/MAPK14 and p38**β**/MAPK11 in spinal cord is enriched in ventral motor neurons.**

Data from the Allen Mouse Spinal Cord Atlas (<http://mousespinal.brain-map.org/>) shows expression of p38 MAPK α and β isoforms of the lumbar spinal cord of an adult mouse [[1](#_ENREF_1)]. Based on location, Nissl staining and somal size, the large cells in the ventral horn are identified as alpha motor neuron cell bodies (see arrowheads for examples). The panels are **(a)** *in situ* hybridization of a section of lumbar spinal cord from an adult mouse showing that distribution of p38α/MAPK14 mRNA is enriched in the cytoplasm of large neurons, particularly in motor neurons of the ventral horn; and **(b)** expression mask derived from the in situ data shows differential expression of the target gene (p38α/MAPK14) with black reflecting no detectable expression, blue showing low expression with green and yellow representing increasing levels of expression. **(c)** *In situ* hybridization of a section of lumbar spinal cord in an adult mouse showing that p38β/MAPK11 mRNA also exhibits higher expression in large cells in the ventral horn, presumptive alpha motor neurons; and **(d)** expression mask showing differential expression of the target gene (p38β/MAPK1). Both p38α and p38β are preferentially expressed in motor neurons.

**Figure S3. Recombinant p38**α **directly phosphorylated both recombinant KHC and immunoprecipitated endogenous brain KHC**.

**(a)** Recombinant p38α was incubated in the presence (+) or absence (–) of KHC (KHC584) recombinant protein. An autoradiogram shows incorporation of ^32^P into KHC584 (*) and autophosphorylated JNK. **(b)**, Recombinant p38α was incubated with immunoprecipitated, endogenous mouse brain kinesin-1. The autoradiogram (^32^P) shows increased phosphorylation of KHC. The accompanying western blot (WB) shows equal amounts of immunoprecipitated KHC in each condition.

**Figure S4.** **Mass spectrometry analysis of kinesin-1 phosphorylation by p38α**.

(**a**) Diagram of mass spectrometry procedures for the analysis of kinesin-1 phosphorylation by p38α showing the path that a protein sample follows during High Performance Liquid Chromatography Mass spectrometry analysis (HPLC-MS. Peptides generated by trypsin treatment of samples are first resolved by a reversed phase column. After peptides elute from the column, ions for mass spectrometry analysis are generated by Electrospray Ionization (ESI). Once peptides enter the mass spectrometer, the most abundant ions are individually selected and captured to go under Collision Induced Dissociation (CID), which yields a collection of shorter sequences for peptide identification. The output of each individual peptide analysis is a mass spectrum that is analyzed by bioinformatics to match to a known protein in the database for protein identification [[9](#_ENREF_9)]. (**b**) Actual mass spectrum of the KIF5c 174-188 phosphopeptide. The graph shows the output mass spectrum, obtained from the mass spectrometer, for one of the identified peptides of the KIF5c protein. The graph plots ion intensity versus mass to charge ion ratio (M/Z) for ***b+*** (red) and ***y+*** (blue) ions that are the direct (N to C terminus) and reverse (C to N terminus) ion series obtained during CID. The identified amino acids peptide sequence for this spectrum is shown in the upper right of the spectrum.

**Figure S5.** **p38α phosphorylation sites on kinesin-1.**

**(a)** The table shows the identified phosphopeptides in the KIF5c rat sequence from recombinant KIF5c phosphorylated by p38α in vitro. From left to right, the table shows the protein ID entry for the database utilized in protein identification analysis; the sequence of the identified phosphopeptide; the mass to ion charge ratio that corresponds unequivocally to that ion or peptide; peptide position in the sequences of the protein (KIF5c) given by the position of the amino (N terminus) and carboxyl (C terminus) amino acid residue; and the last column indicates the position of the actual phosphorylated residue. Of these peptides, the only sites conserved between human, mouse and squid kinesin-1 protein were S175/S176 in peptide 173-190 (shown in red). **(b)** Several parameters are shown for the identified phosphopeptides. From left to right: File name of the mass spectrum obtained from the mass spectrometer for the peptide, total mass of the ion or peptide, x correlation (XCorr) and delta correlation value (dCn) for each identified peptide. These two parameters emerge from the bioinformatic data analysis after mass spectrometry. These values are used to decide whether a peptide should be reported or not. The cut off values were specified in materials and methods (see above). The next two columns indicate the number of identified peptide during CID and the total number of theoretical ions. Finally, the protein name entry in the database and the peptide sequence are given.

**Figure S6. DVD Peptide Prevents Inhibition of FAT by mutant SOD1.**

Co-perfusion of G93A-SOD1 with DVD peptide **(a)**, but not with a control DVD peptide **(b)** prevents inhibition of FAT induced by G93A-SOD1. DVD peptide prevents activation of MKKs by some MKKKs (n = number of axoplasms), whereas DVD control peptide does not [[10](#_ENREF_10)]. These data suggest that the activation of p38 and the inhibition of FAT induced by G93A-SOD1 involve activation of one or more MAPKKKs that require the DVD docking motif for activation of downstream kinases.

**Figure S7.** **Inhibition of p38 attenuates mSOD1-induced apoptosis.**

Expression of mutant SOD1 in N2A cells has a very modest effect on cell viability that is greatly enhanced by challenge with cyclosporine A (CsA). Stably transfected N2A cells expressing WT-SOD1 (WT-SOD1, left panels) or G85R-SOD1 (mSOD1, right panels) were incubated with various concentrations of CsA, (0 to 14 µg/ml) in the presence (+) or absence (-) of the p38 inhibitor SB203580 (10µM). **(a)** LDH toxicity assays show a dose-dependent increase in CsA-induced cytotoxicity on both WT-SOD1 and mSOD1 cell lines. However, the toxic effect of CsA is more pronounced in cells expressing mSOD1 (hatched red bars), compared to cells expressing WT-SOD1 (black striped bars) [[5](#_ENREF_5)]. Remarkably, treatment of mSOD1 N2A cells with the p38 inhibitor SB203580 significantly attenuated cell death at 0, 3.5 and 7µg/ml CsA (solid bars). In contrast, SB203580 reduced CsA-induced toxicity at 7µg/ml, but not 3.5µg/ml (solid bars) in WT-SOD1 N2A cells. Data represent the mean ± SEM % cytotoxicity for n=8 wells (* p <0.0001). **(b)** Caspase-Glo assays confirmed and extended results in **a**, showing that CsA induced the activation of the pro-apoptotic caspases 3 and 7 in mSOD1 N2A cells (red hatched bars) to a greater extent than WT-SOD1 N2A cells (black striped bars). Treatment of mSOD1 N2A cells with SB203580 (solid bars) significantly attenuated caspase 3/7 activation, whereas WT-SOD1 cells exhibited similar caspase activity levels in the presence (solid bars) and absence (striped black bars) of SB203580. Data represent the mean ± SEM luminescence signal for treated cells relative to untreated cells for n = 4 wells (* p <0.0001). These results suggest p38 activity contributes to the increased vulnerability of mSOD1 N2A cells to CsA-induced cell death.

**Figure S8. Inhibition of conventional kinesin-based motility induced by pathogenic SOD1.**

Our results showing increased activation and phosphorylation of p38 by mSOD1 polypeptides suggest that these pathogenic mSOD1 polypeptides activate specific MAPKKKs and MAPKKs (dashed arrow) upstream of p38 (Fig. 9 and see [[11](#_ENREF_11)]). Activation of axonal p38 would lead to phosphorylation of kinesin-1, neurofilaments (NFs) and likely other axonal substrates. Data in this work indicates that phosphorylation of kinesin-1 by p38 inhibits translocation of conventional kinesin along microtubules. Reductions in the delivery of critical axonal cargoes by conventional kinesin, (such as synaptic vesicle precursors and organelles containing neurotrophin receptors) would result in impaired synaptic function and dying-back degeneration of neurons [[12](#_ENREF_12)]. In addition, increased p38 activation in neuronal cell bodies would be expected to promote alterations in the activity of various transcription factors (i.e., ATF-2 and c-Jun, among others), consistent with reports of transcriptional changes and activation of apoptosis induced by pathogenic SOD1 expression.

**SUPPLEMENTAL REFERENCES**

1. Ng L, Bernard A, Lau C, Overly CC, Dong HW, et al. (2009) An anatomic gene expression atlas of the adult mouse brain. Nat Neurosci 12: 356-362.

2. Pasinelli P, Belford ME, Lennon N, Bacskai BJ, Hyman BT, et al. (2004) Amyotrophic lateral sclerosis-associated SOD1 mutant proteins bind and aggregate with Bcl-2 in spinal cord mitochondria. Neuron 43: 19-30.

3. Pasinelli P, Borchelt DR, Houseweart MK, Cleveland DW, Brown RH, Jr. (1998) Caspase-1 is activated in neural cells and tissue with amyotrophic lateral sclerosis-associated mutations in copper-zinc superoxide dismutase. Proc Natl Acad Sci U S A 95: 15763-15768.

4. Kim EK, Choi EJ (2010) Pathological roles of MAPK signaling pathways in human diseases. Biochim Biophys Acta 1802: 396-405.

5. Maxwell MM, Pasinelli P, Kazantsev AG, Brown RH, Jr. (2004) RNA interference-mediated silencing of mutant superoxide dismutase rescues cyclosporin A-induced death in cultured neuroblastoma cells. Proc Natl Acad Sci U S A 101: 3178-3183.

6. Deboer SR, You Y, Szodorai A, Kaminska A, Pigino G, et al. (2008) Conventional Kinesin Holoenzymes Are Composed of Heavy and Light Chain Homodimers. Biochemistry 47: 4535-4543.

7. Morfini GA, You YM, Pollema SL, Kaminska A, Liu K, et al. (2009) Pathogenic huntingtin inhibits fast axonal transport by activating JNK3 and phosphorylating kinesin. Nat Neurosci 12: 864-871.

8. Bolte S, Cordelieres FP (2006) A guided tour into subcellular colocalization analysis in light microscopy. J Microsc 224: 213-232.

9. Steen H, Mann M (2004) The ABC's (and XYZ's) of peptide sequencing. Nat Rev Mol Cell Biol 5: 699-711.

10. Takekawa M, Tatebayashi K, Saito H (2005) Conserved docking site is essential for activation of mammalian MAP kinase kinases by specific MAP kinase kinase kinases. Mol Cell 18: 295-306.

11. Song Y, Nagy M, Ni W, Tyagi NK, Fenton WA, et al. (2013) Molecular chaperone Hsp110 rescues a vesicle transport defect produced by an ALS-associated mutant SOD1 protein in squid axoplasm. Proc Natl Acad Sci U S A 110: 5428-5433.

12. Morfini GA, Burns M, Binder LI, Kanaan NM, LaPointe N, et al. (2009) Axonal transport defects in neurodegenerative diseases. J Neurosci 29: 12776-12786.
