# Supplementary figures and images for: Inhibition of Fast Axonal Transport by Pathogenic SOD1 Involves Activation of p38 MAP Kinase
Source: PLoS One. 2013 Jun 12;8(6):e65235. doi: 10.1371/journal.pone.0065235 (PMC3680447; doi:10.1371/journal.pone.0065235)

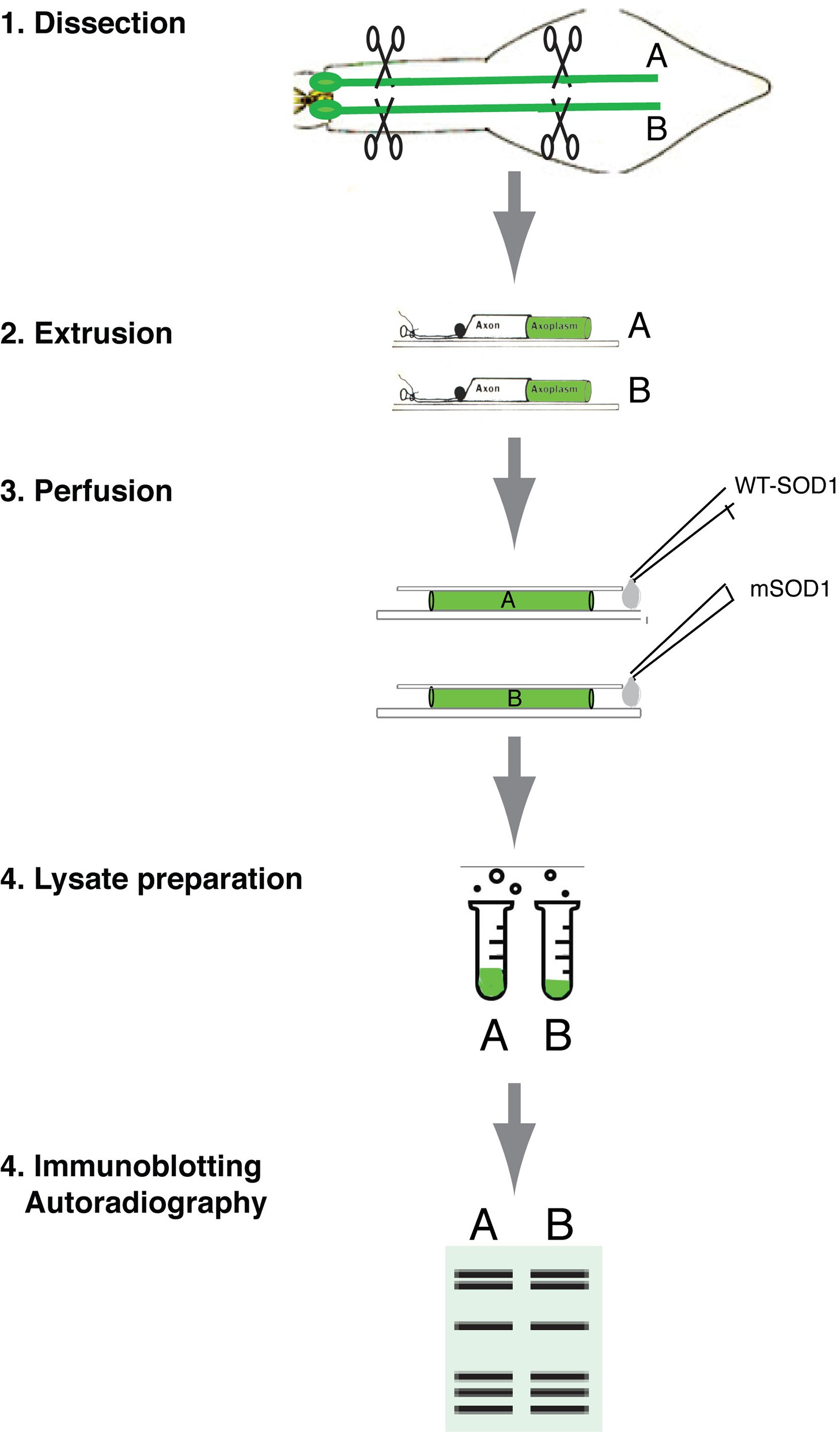

Supplement: Figure S1 — Outline of metabolic labeling experiments and immunobloting analysis in isolated squid axoplasm. Two giant axons were dissected from the same squid (“sister” axons), extruded, placed on glass coverslips, and incubated with recombinant SOD1 proteins. One axon was perfused with WT-SOD1, whereas the contralateral axon was perfused with pathogenic SOD1. For metabolic labeling experiments in Figure 2, an aliquot of radiolabelled 32P-ATP was added to each axoplasm. After a 50-minute incubation, axons were lysed and processed for autoradiography (Fig. 2) or immunoblotting (Fig. 4). (TIF) [file pone.0065235.s002.tif]

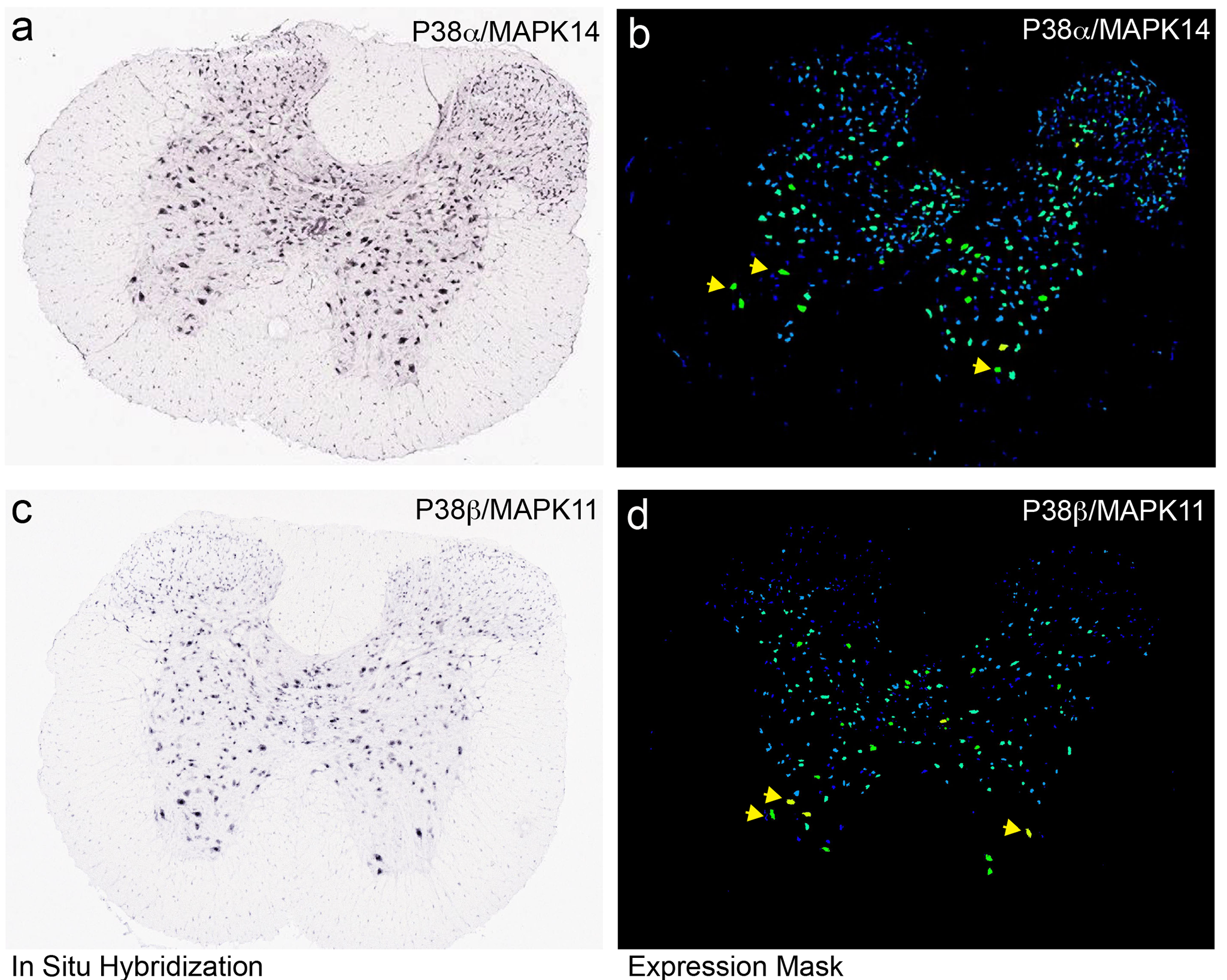

Supplement: Figure S2 — Expression of p38α/MAPK14 and p38β/MAPK11 in spinal cord is enriched in ventral motor neurons. Data from the Allen Mouse Spinal Cord Atlas (http://mousespinal.brain-map.org/) shows expression of p38 MAPK α and β isoforms of the lumbar spinal cord of an adult mouse. Based on location, Nissl staining and somal size, the large cells in the ventral horn are identified as alpha motor neuron cell bodies (see arrowheads for examples). The panels are (a) in situ hybridization of a section of lumbar spinal cord from an adult mouse showing that distribution of p38α/MAPK14 mRNA is enriched in the cytoplasm of large neurons, particularly in motor neurons of the ventral horn; and (b) expression mask derived from the in situ data shows differential expression of the target gene (p38α/MAPK14) with black reflecting no detectable expression, blue showing low expression with green and yellow representing increasing levels of expression. (c) In situ hybridization of a section of lumbar spinal cord in an adult mouse showing that p38β/MAPK11 mRNA also exhibits higher expression in large cells in the ventral horn, presumptive alpha motor neurons; and (d) expression mask showing differential expression of the target gene (p38β/MAPK1). Both p38α and p38β are preferentially expressed in motor neurons. (TIF) [file pone.0065235.s003.tif]

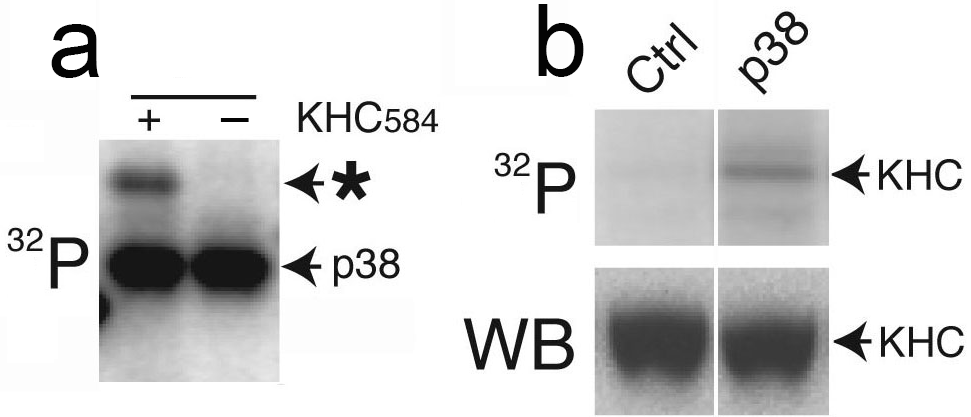

Supplement: Figure S3 — Recombinant p38α directly phosphorylated both recombinant KHC and immunoprecipitated endogenous brain KHC. (a), Recombinant p38α was incubated in the presence (+) or absence (–) of KHC (KHC584) recombinant protein. An autoradiogram shows incorporation of 32P into KHC584 (*) and autophosphorylated JNK. (b), Recombinant p38α was incubated with immunoprecipitated, endogenous mouse brain kinesin-1. The autoradiogram (32P) shows increased phosphorylation of KHC. The accompanying western blot (WB) shows equal amounts of immunoprecipitated KHC in each condition. (TIF) [file pone.0065235.s004.tif]

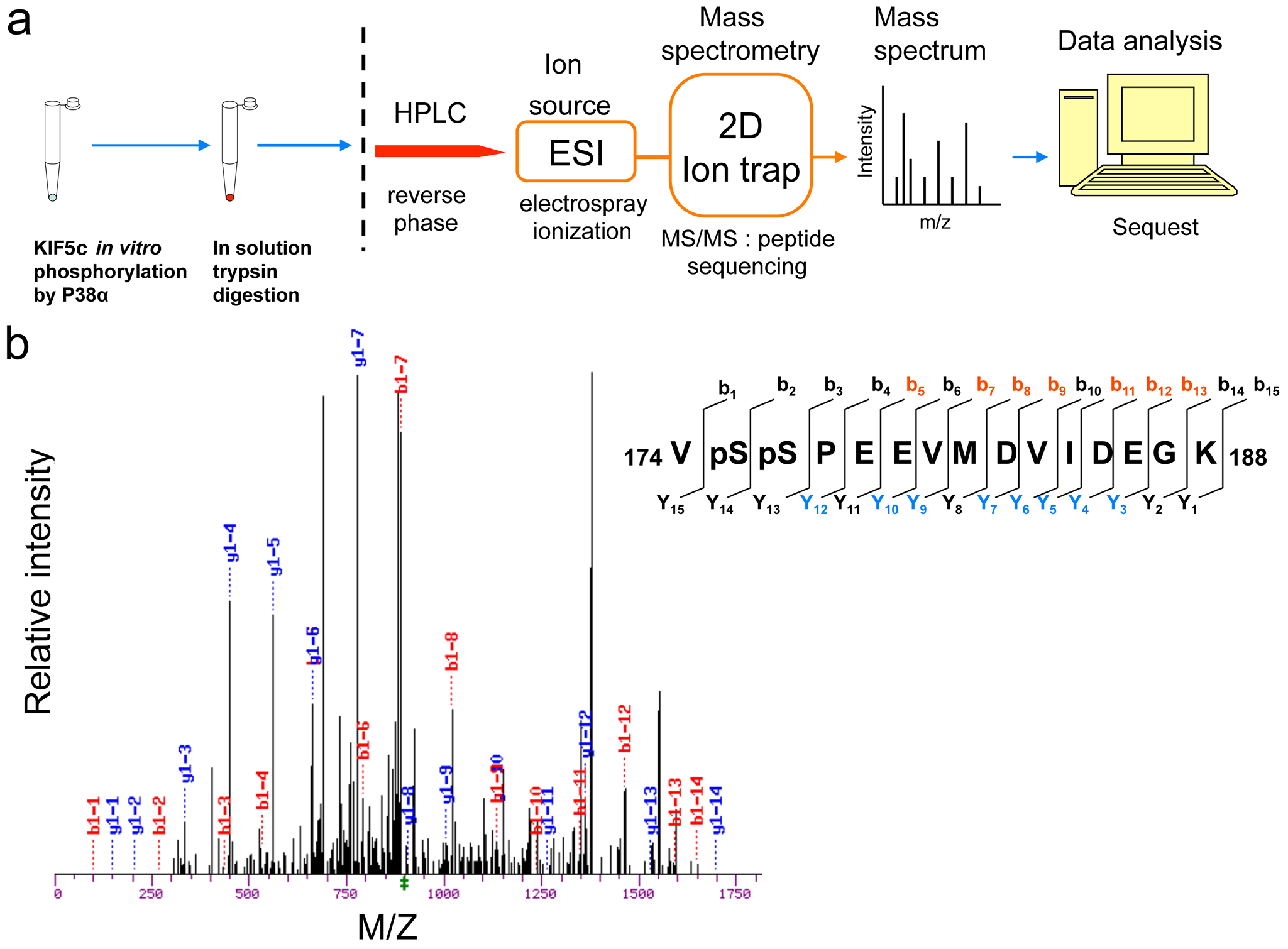

Supplement: Figure S4 — Mass spectrometry analysis of kinesin-1 phosphorylation by p38α. (a) Diagram of mass spectrometry procedures for the analysis of kinesin-1 phosphorylation by p38α showing the path that a protein sample follows during High Performance Liquid Chromatography Mass spectrometry analysis (HPLC-MS. Peptides generated by trypsin treatment of samples are first resolved by a reversed phase column. After peptides elute from the column, ions for mass spectrometry analysis are generated by Electrospray Ionization (ESI). Once peptides enter the mass spectrometer, the most abundant ions are individually selected and captured to go under Collision Induced Dissociation (CID), which yields a collection of shorter sequences for peptide identification. The output of each individual peptide analysis is a mass spectrum that is analyzed by bioinformatics to match to a known protein in the database for protein identification. (b) Actual mass spectrum of the KIF5c 174–188 phosphopeptide. The graph shows the output mass spectrum, obtained from the mass spectrometer, for one of the identified peptides of the KIF5c protein. The graph plots ion intensity versus mass to charge ion ratio (M/Z) for b+ (red) and y+ (blue) ions that are the direct (N to C terminus) and reverse (C to N terminus) ion series obtained during CID. The identified amino acids peptide sequence for this spectrum is shown in the upper right of the spectrum. (TIF) [file pone.0065235.s005.tif]

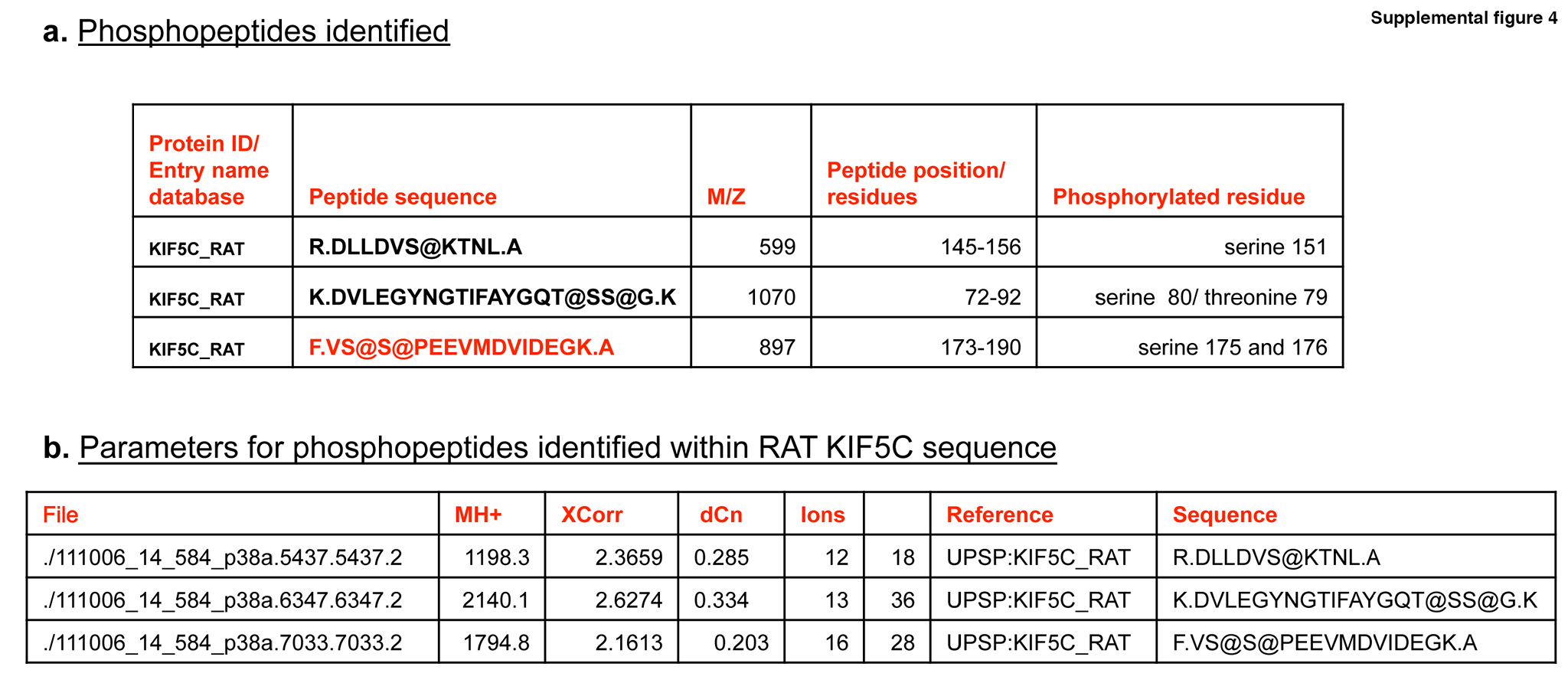

Supplement: Figure S5 — p38α phosphorylation sites on kinesin-1. (a) The table shows the identified phosphopeptides in the KIF5c rat sequence from recombinant KIF5c phosphorylated by p38α in vitro. From left to right, the table shows the protein ID entry for the database utilized in protein identification analysis; the sequence of the identified phosphopeptide; the mass to ion charge ratio that corresponds unequivocally to that ion or peptide; peptide position in the sequences of the protein (KIF5c) given by the position of the amino (N terminus) and carboxyl (C terminus) amino acid residue; and the last column indicates the position of the actual phosphorylated residue. Of these peptides, the only sites conserved between human, mouse and squid kinesin-1 protein were S175/S176 in peptide 173–190 (shown in red). (b) Several parameters are shown for the identified phosphopeptides. From left to right: File name of the mass spectrum obtained from the mass spectrometer for the peptide, total mass of the ion or peptide, x correlation (XCorr) and delta correlation value (dCn) for each identified peptide. These two parameters emerge from the bioinformatic data analysis after mass spectrometry. These values are used to decide whether a peptide should be reported or not. The cut off values were specified in materials and methods (see above). The next two columns indicate the number of identified peptide during CID and the total number of theoretical ions. Finally, the protein name entry in the database and the peptide sequence are given. (TIF) [file pone.0065235.s006.tif]

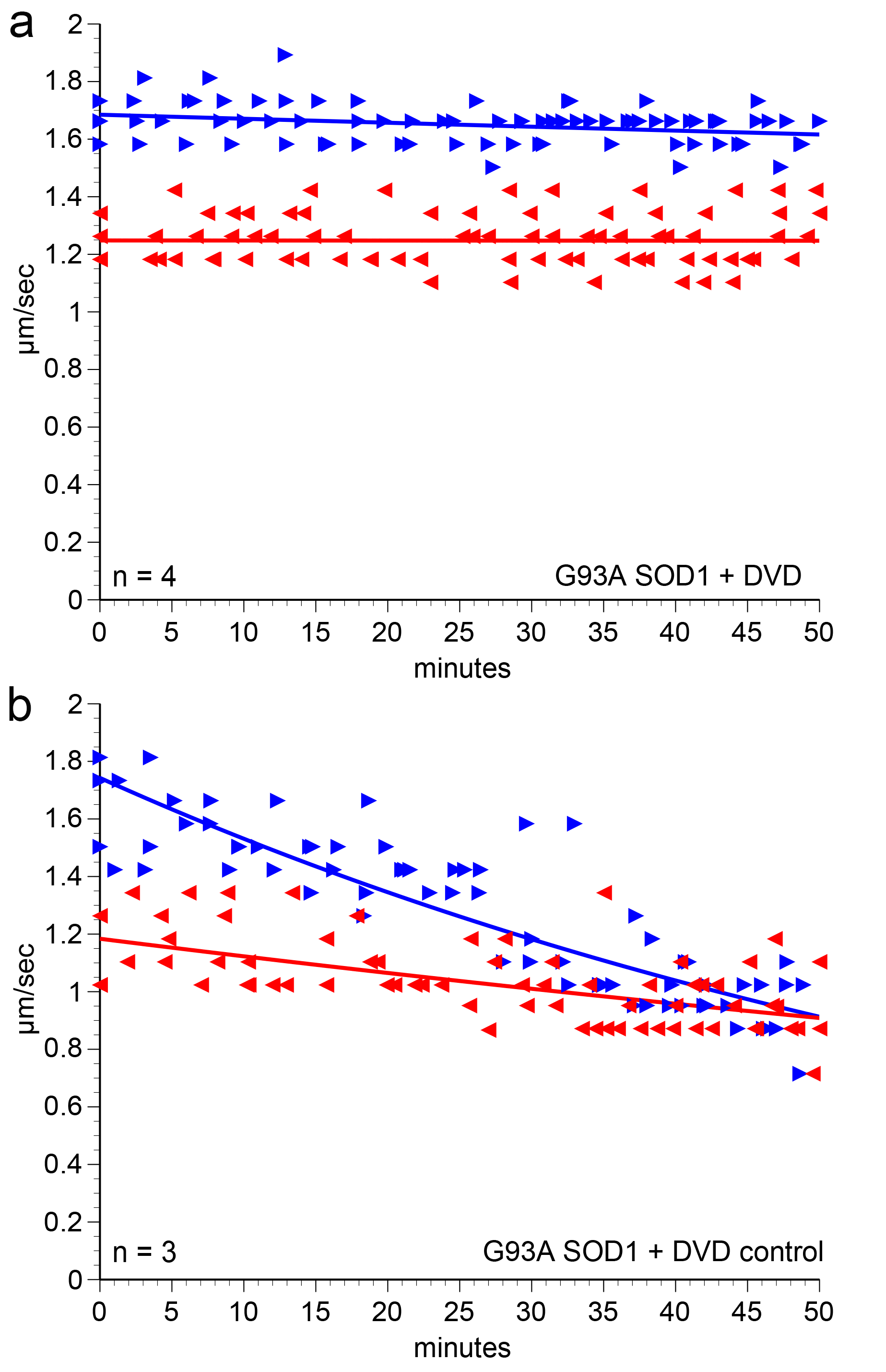

Supplement: Figure S6 — DVD Peptide Prevents Inhibition of FAT by mutant SOD1. Co-perfusion of G93A-SOD1 with DVD peptide (a), but not with a control DVD peptide (b) prevents inhibition of FAT induced by G93A-SOD1. DVD peptide prevents activation of MKKs by some MKKKs (n = number of axoplasms), whereas DVD control peptide does not. These data suggest that the activation of p38 and the inhibition of FAT induced by G93A-SOD1 involve activation of one or more MAPKKKs that require the DVD docking motif for activation of downstream kinases. (TIF) [file pone.0065235.s007.tif]

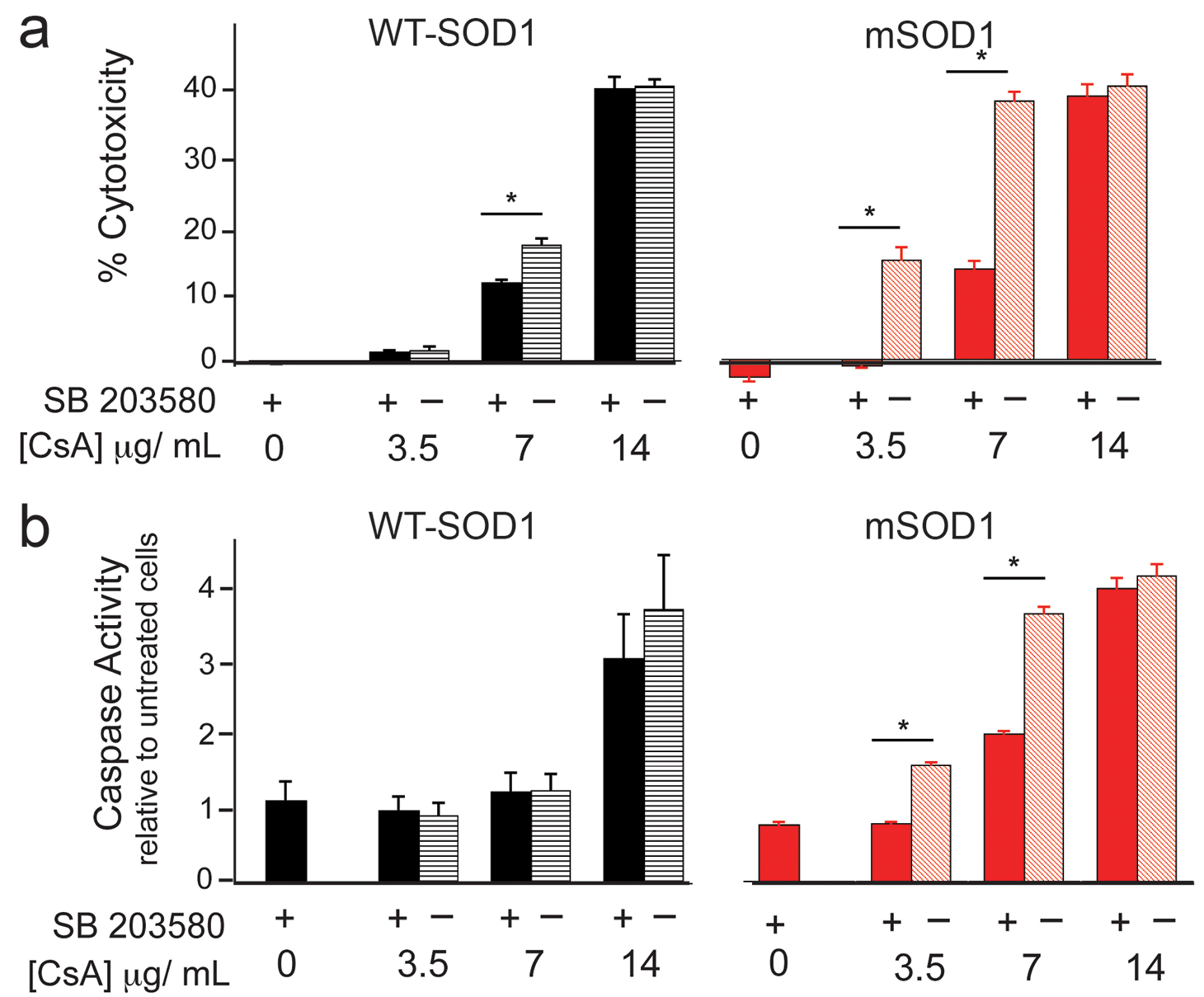

Supplement: Figure S7 — Inhibition of p38 attenuates mSOD1-induced apoptosis. Expression of mutant SOD1 in N2A cells has a very modest effect on cell viability that is greatly enhanced by challenge with cyclosporine A (CsA). Stably transfected N2A cells expressing WT-SOD1 (WT-SOD1, left panels) or G85R-SOD1 (mSOD1, right panels) were incubated with various concentrations of CsA, (0 to 14 µg/ml) in the presence (+) or absence (–) of the p38 inhibitor SB203580 (10 µM). (a) LDH toxicity assays show a dose-dependent increase in CsA-induced cytotoxicity on both WT-SOD1 and mSOD1 cell lines. However, the toxic effect of CsA is more pronounced in cells expressing mSOD1 (hatched red bars), compared to cells expressing WT-SOD1 (black striped bars). Remarkably, treatment of mSOD1 N2A cells with the p38 inhibitor SB203580 significantly attenuated cell death at 0, 3.5 and 7 µg/ml CsA (solid bars). In contrast, SB203580 reduced CsA-induced toxicity at 7 µg/ml, but not 3.5 µg/ml (solid bars) in WT-SOD1 N2A cells. Data represent the mean ± SEM % cytotoxicity for n = 8 wells (* p<0.0001). (b) Caspase-Glo assays confirmed and extended results in a, showing that CsA induced the activation of the pro-apoptotic caspases 3 and 7 in mSOD1 N2A cells (red hatched bars) to a greater extent than WT-SOD1 N2A cells (black striped bars). Treatment of mSOD1 N2A cells with SB203580 (solid bars) significantly attenuated caspase 3/7 activation, whereas WT-SOD1 cells exhibited similar caspase activity levels in the presence (solid bars) and absence (striped black bars) of SB203580. Data represent the mean ± SEM luminescence signal for treated cells relative to untreated cells for n = 4 wells (* p<0.0001). These results suggest p38 activity contributes to the increased vulnerability of mSOD1 N2A cells to CsA-induced cell death. (TIF) [file pone.0065235.s008.tif]

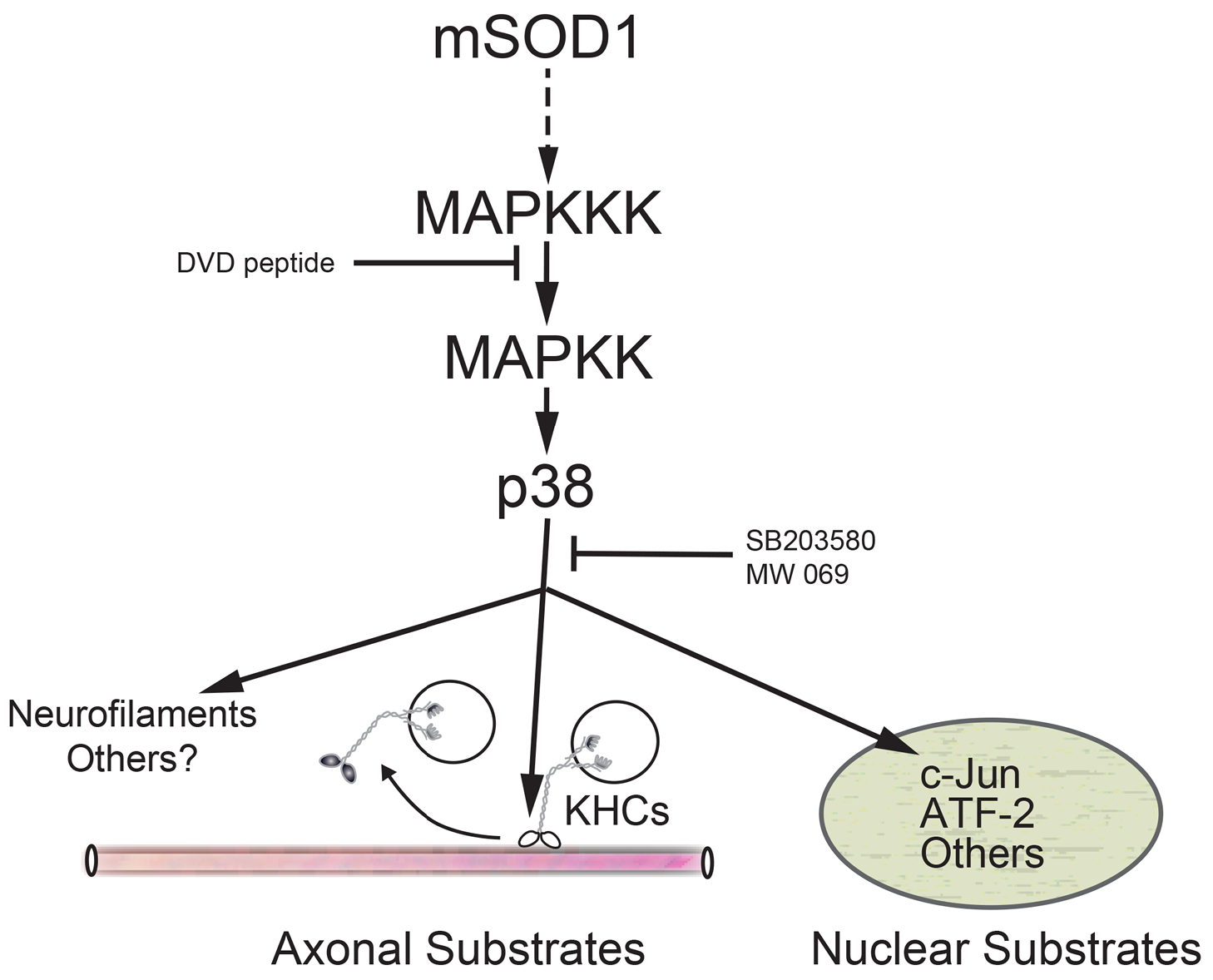

Supplement: Figure S8 — Inhibition of conventional kinesin-based motility induced by pathogenic SOD1. Our results showing increased activation and phosphorylation of p38 by mSOD1 polypeptides suggest that these pathogenic mSOD1 polypeptides activate specific MAPKKKs and MAPKKs (dashed arrow) upstream of p38 (Fig. 9). Activation of axonal p38 would lead to phosphorylation of kinesin-1, neurofilaments (NFs) and likely other axonal substrates. Data in this work indicates that phosphorylation of kinesin-1 by p38 inhibits translocation of conventional kinesin along microtubules. Reductions in the delivery of critical axonal cargoes by conventional kinesin, (such as synaptic vesicle precursors and organelles containing neurotrophin receptors) would result in impaired synaptic function and dying-back degeneration of neurons In addition, increased p38 activation in neuronal cell bodies would be expected to promote alterations in the activity of various transcription factors (i.e., ATF-2 and c-Jun, among others), consistent with reports of transcriptional changes and activation of apoptosis induced by pathogenic SOD1 expression. (TIF) [file pone.0065235.s009.tif]
